# EVC-EVC2 complex stability and ciliary targeting are regulated by modification with ubiquitin and SUMO

**DOI:** 10.3389/fcell.2023.1190258

**Published:** 2023-07-27

**Authors:** Pablo Barbeito, Raquel Martin-Morales, Adrian Palencia-Campos, Juan Cerrolaza, Celia Rivas-Santos, Leticia Gallego-Colastra, Jose Antonio Caparros-Martin, Carolina Martin-Bravo, Ana Martin-Hurtado, Laura Sánchez-Bellver, Gemma Marfany, Victor L. Ruiz-Perez, Francesc R. Garcia-Gonzalo

**Affiliations:** ^1^ Departamento de Bioquímica, Facultad de Medicina, Universidad Autónoma de Madrid (UAM), Madrid, Spain; ^2^ Instituto de Investigaciones Biomédicas “Alberto Sols” (IIBM), Consejo Superior de Investigaciones Científicas (CSIC)-UAM, Madrid, Spain; ^3^ CIBER de Enfermedades Raras (CIBERER), Instituto de Salud Carlos III (ISCIII), Madrid, Spain; ^4^ Instituto de Investigación del Hospital Universitario de La Paz (IdiPAZ), Madrid, Spain; ^5^ Departament de Genètica, Microbiologia i Estadística, Universitat de Barcelona, Barcelona, Spain; ^6^ Institut de Biomedicina—Institut de Recerca Sant Joan de Déu (IBUB-IRSJD), Universitat de Barcelona, Barcelona, Spain; ^7^ DBGen Ocular Genomics, Barcelona, Spain

**Keywords:** Ellis van Creveld syndrome, Weyers acrofacial dysostosis, ciliopathy, cilia, Hedgehog signaling, ubiquitin, SUMO, interactome

## Abstract

Ellis van Creveld syndrome and Weyers acrofacial dysostosis are two rare genetic diseases affecting skeletal development. They are both ciliopathies, as they are due to malfunction of primary cilia, microtubule-based plasma membrane protrusions that function as cellular antennae and are required for Hedgehog signaling, a key pathway during skeletal morphogenesis. These ciliopathies are caused by mutations affecting the EVC-EVC2 complex, a transmembrane protein heterodimer that regulates Hedgehog signaling from inside primary cilia. Despite the importance of this complex, the mechanisms underlying its stability, targeting and function are poorly understood. To address this, we characterized the endogenous EVC protein interactome in control and *Evc*-null cells. This proteomic screen confirmed EVC’s main known interactors (EVC2, IQCE, EFCAB7), while revealing new ones, including USP7, a deubiquitinating enzyme involved in Hedgehog signaling. We therefore looked at EVC-EVC2 complex ubiquitination. Such ubiquitination exists but is independent of USP7 (and of USP48, also involved in Hh signaling). We did find, however, that monoubiquitination of EVC-EVC2 cytosolic tails greatly reduces their protein levels. On the other hand, modification of EVC-EVC2 cytosolic tails with the small ubiquitin-related modifier SUMO3 has a different effect, enhancing complex accumulation at the EvC zone, immediately distal to the ciliary transition zone, possibly via increased binding to the EFCAB7-IQCE complex. Lastly, we find that EvC zone targeting of EVC-EVC2 depends on two separate EFCAB7-binding motifs within EVC2’s Weyers-deleted peptide. Only one of these motifs had been characterized previously, so we have mapped the second herein. Altogether, our data shed light on EVC-EVC2 complex regulatory mechanisms, with implications for ciliopathies.

## 1 Introduction

Ellis van Creveld syndrome (EvC; MIM 225500) is a rare autosomal recessive chondroectodermal dysplasia affecting around 1 in 60,000 live births. The most common manifestations of this condition include short stature, bilateral postaxial polydactyly, dysplastic teeth and nails, and cardiac malformations. In most cases, EvC is due to mutations in one of two back-to-back genes on chromosome 4: the *EVC* and *EVC2* genes. These genes encode two single-pass transmembrane proteins, EVC and EVC2, which form a heterodimeric complex that stabilizes both proteins. This complex localizes inside primary cilia, where it is involved in Hedgehog (Hh) signaling. The Hh pathway plays essential roles during embryogenesis and later life, and is a key regulator of many stem cell populations. Mutations disrupting EVC-EVC2 complex integrity disrupt Hh signaling, which in turn leads to the observed manifestations of EvC (Bangs and Anderson, 2017; Blair et al., 2011; Caparros-Martin et al., 2013; D'Asdia et al., 2013; Dorn et al., 2012; Ruiz-Perez and Goodship, 2009; Ruiz-Perez et al., 2000; Ruiz-Perez et al., 2003; Yang et al., 2012).

Weyers acrofacial dysostosis (WAD; MIM 193530) is another rare disorder, similar to EvC but with milder manifestations. Unlike EvC, WAD is inherited dominantly. While EvC is typically caused by mutations disrupting assembly or stability of the EVC-EVC2 complex, thus preventing it from accumulating in cilia, WAD is instead caused by specific deletions affecting up to 43 amino acid (aa) residues at the end of EVC2’s C-terminal cytosolic tail (D'Asdia et al., 2013; Ruiz-Perez and Goodship, 2009). These residues, known as the Weyers peptide (W-peptide), are not needed for complex formation, nor are they needed for ciliary targeting. Instead, they are essential for EVC-EVC2 complex targeting to a ciliary subcompartment known as the EvC zone (Dorn et al., 2012; Caparros-Martin et al., 2013; Pusapati et al., 2014).

Primary cilia are thin microtubule protrusions of the plasma membrane that function as cell type-specific antennae, detecting chemical, mechanical or optical signals in different tissues. In the case of Hh-responding cell types (e.g., chondrocytes or fibroblasts), these antennae are equipped with all the necessary receptors and transducers for Hh signaling. These include proteins like Patched (the Hh ligand receptor), Smoothened (a major effector of the pathway), and the EVC-EVC2 complex (which promotes signal transduction from Smoothened to downstream effectors) (Ruiz-Perez et al., 2007; Ruiz-Perez and Goodship, 2009; Blair et al., 2011; Dorn et al., 2012; Caparros-Martin et al., 2013; Bangs and Anderson, 2017; Reiter and Leroux, 2017).

Primary cilia contain several subcompartments. Their microtubule shaft, or axoneme, emanates from the basal body, a membrane-docked centriole at the ciliary base. The region where the basal body transitions into the axoneme is known as the transition zone, a border region controlling traffic into and out of the cilium. The whole axoneme, including at the transition zone, is covered by a specialized plasma membrane patch known as the ciliary membrane (Garcia-Gonzalo and Reiter, 2012; Garcia-Gonzalo and Reiter, 2017; Reiter and Leroux, 2017).

EVC-EVC2 specifically localize to the membrane at the EvC zone, a ciliary region located immediately distal to the transition zone (Dorn et al., 2012; Caparros-Martin et al., 2013). In WAD, W-peptide deletion causes the EVC-EVC2 complex to localize uniformly throughout the ciliary membrane, rather than accumulating at the EvC zone, and this impairs Hh signaling (Dorn et al., 2012; Caparros-Martin et al., 2013). The mechanism of action of the W-peptide has been resolved in some detail. The EVC-EVC2 complex is recruited to the EvC zone by another complex, the EFCAB7-IQCE complex, and the W-peptide, by interacting with EFCAB7, is essential for this recruitment (Pusapati et al., 2014). Within the W-peptide, a phenylalanine-valine (FV) motif is essential for this interaction (Dorn et al., 2012; Pusapati et al., 2014). However, since some W-peptide deletions affect EvC zone targeting without removing the FV motif, it follows that other motifs within the W-peptide must also be required for such targeting (Caparros-Martin et al., 2013). Indeed, at the end of this work we show that another motif is also needed.

Herein, we start characterizing the EVC interactome in mouse embryonic fibroblasts (MEFs). Besides known interactors, we identified new ones that may prove important for EVC-EVC2 complex function. One of them is USP7, a deubiquitinating enzyme involved in Hh signaling (Zhou et al., 2015; Zhan et al., 2017; Bhattacharya et al., 2018). We also found that EVC-EVC2 undergo ubiquitination, but this does not appear to depend on USP7. We found instead that monoubiquitination of EVC-EVC2 cytosolic tails strongly lowers their levels, whereas their sumoylation enhances EFCAB7 binding and EvC zone targeting of the complex.

## 2 Results

### 2.1 Proteomic identification of the endogenous EVC interactome

**FIGURE 1:**
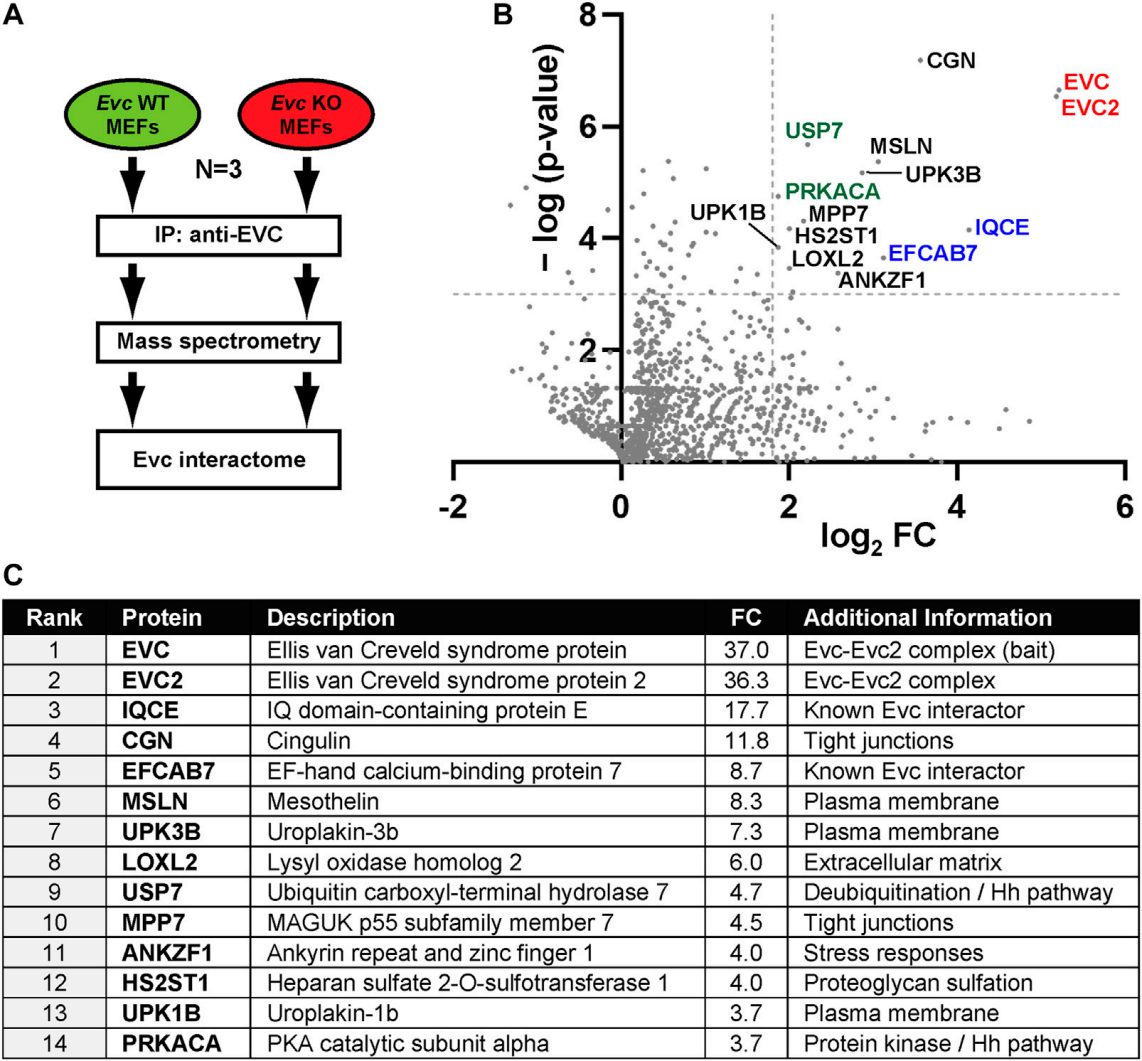
The EVC interactome. **(A)** Schematic of the interactomic experiments. Lysates from *Evc*-WT and *Evc*-KO mouse embryonic fibroblasts (MEFs) were subjected to immunoprecipitation (IP) using antibodies against the endogenous mouse EVC protein. Immunoprecipitated proteins were then analyzed by tandem mass spectrometry. The experiment was repeated three independent times (N = 3). Data were then analyzed to identify specific EVC interactors (enriched in *Evc*-WT relative to *Evc*-KO samples). **(B)** Volcano plot of the EVC interactome. For each identified protein, the *x*-axis represents the binary logarithm of the fold change (FC) between *Evc*-WT and *Evc*-KO samples, whereas the *y*-axis shows the negative decimal logarithm of the *p*-value from a Student’s t-test (two-tailed, homoscedastic), comparing *Evc*-WT to *Evc*-KO. Dashed lines indicate cutoff values at *p* = 0.001 and FC = 3.5. Protein names of all main hits are indicated. Among these are EVC-EVC2 (red), their known interactors IQCE-EFCAB7 (blue), and Hedgehog pathway regulators PRKACA and USP7 (green). All other hits are indicated in black. **(C)** Table showing the main EVC interactome hits, ranked by FC. See methods section and supplementary material for more details.

To gain deeper knowledge into the function of the EVC-EVC2 complex, we used proteomics to identify EVC interactors, which we pulled down using a previously described mouse monoclonal anti-EVC antibody (Pacheco et al., 2012). We did this in fibroblasts, a cell type where the EVC-EVC2 complex localizes to cilia and is essential for Hedgehog signaling (Dorn et al., 2012; Yang et al., 2012; Caparros-Martin et al., 2013; Pusapati et al., 2014). More specifically, we used both *Evc* ^+/+^ and *Evc* ^−/−^ MEFs, which we reported previously (Ruiz-Perez et al., 2007; Caparros-Martin et al., 2013). The *Evc* ^−/−^ MEFs functioned as the negative control of the experiment, which we repeated *n* = 3 independent times. Using this approach, we identified a number of proteins that were pulled down specifically in *Evc* ^+/+^ but not *Evc* ^−/−^ MEFs. The main results of these experiments are featured in Figure 1, and the raw data and quantitative analyses are available in Supplementary Data File 1.

As expected, the two most enriched proteins in the wild type MEF IPs were EVC itself, the bait, and EVC2, its interacting partner in the EVC-EVC2 complex (Figure 1). The fact that both proteins were pulled down in virtually identical amounts in all three experiments supports previous data showing that both proteins form a heterodimeric complex and stabilize each other (Blair et al., 2011; Caparros-Martin et al., 2013). The EVC-EVC2 complex is recruited to the ciliary base by interacting with the IQCE-EFCAB7 complex (Pusapati et al., 2014). Accordingly, IQCE and EFCAB7 also featured prominently in our EVC interactome (Figure 1). Thus, our approach proved its validity by readily identifying well-known EVC interactors.

In addition, this approach revealed putative EVC interactors that may hold important clues to EVC-EVC2 complex functions (Figure 1; Supplementary Data File 1). Consistent with EVC-EVC2’s transmembrane nature, most of the shortlisted hits include plasma membrane-associated and/or secretory pathway proteins (MSLN, UPK1B, UPK3b, LOXL2, HS2ST1, CGN) (Figure 1C) (UniProt, 2023). Besides EVC-EVC2 and IQCE-EFCAB7, the shortlisted hits include ten proteins with the following reported functions: i) Cingulin (CGN) and MPP7 are involved in tight junction biology (Bohl et al., 2007; Stucke et al., 2007; Van Itallie and Anderson, 2014); ii) Uroplakins (UPK1B-UPK3B) form a complex controlling epithelial permeability (Wu et al., 2009); iii) LOXL2 and HS2ST1 affect extracellular matrix composition (Teixeira et al., 2020; Liburkin-Dan et al., 2022); iv) ANKZF1 releases peptidyl-tRNA complexes from stalled ribosomes and protects cells from stress (van Haaften-Visser et al., 2017; Kuroha et al., 2018; Verma et al., 2018); v) Mesothelin (MSLN) is a transmembrane protein whose ectodomain is shed and has signaling functions (Koyama et al., 2017); vi) PRKACA is the catalytic subunit α of protein kinase A (Turnham and Scott, 2016); and vii) USP7 is a deubiquitinating enzyme with multiple substrates, including among others p53, PTEN and β-catenin (Bhattacharya et al., 2018). The biological significance of all these interactions remains to be addressed.

Given the role of EVC-EVC2 in Hh signaling, we paid special attention to interactors known to regulate this pathway. Besides IQCE-EFCAB7, this includes PRKACA, a negative Hh pathway regulator whose hypermorphic mutations cause an EvC-like syndrome (Bangs and Anderson, 2017; Palencia-Campos et al., 2020; Happ et al., 2022), and USP7 (ubiquitin carboxyl-terminal hydrolase 7), whose many deubiquitination substrates include the GLI transcription factors, the main mediators of Hh pathway output (Zhou et al., 2015; Bhattacharya et al., 2018). This raised the possibility that the EVC-EVC2 complex undergoes ubiquitination, which might be counteracted by USP7 with potentially important functional consequences. We next set about testing these hypotheses.

### 2.2 EVC-EVC2 undergo ubiquitination in a USP7-independent manner

**FIGURE 2:**
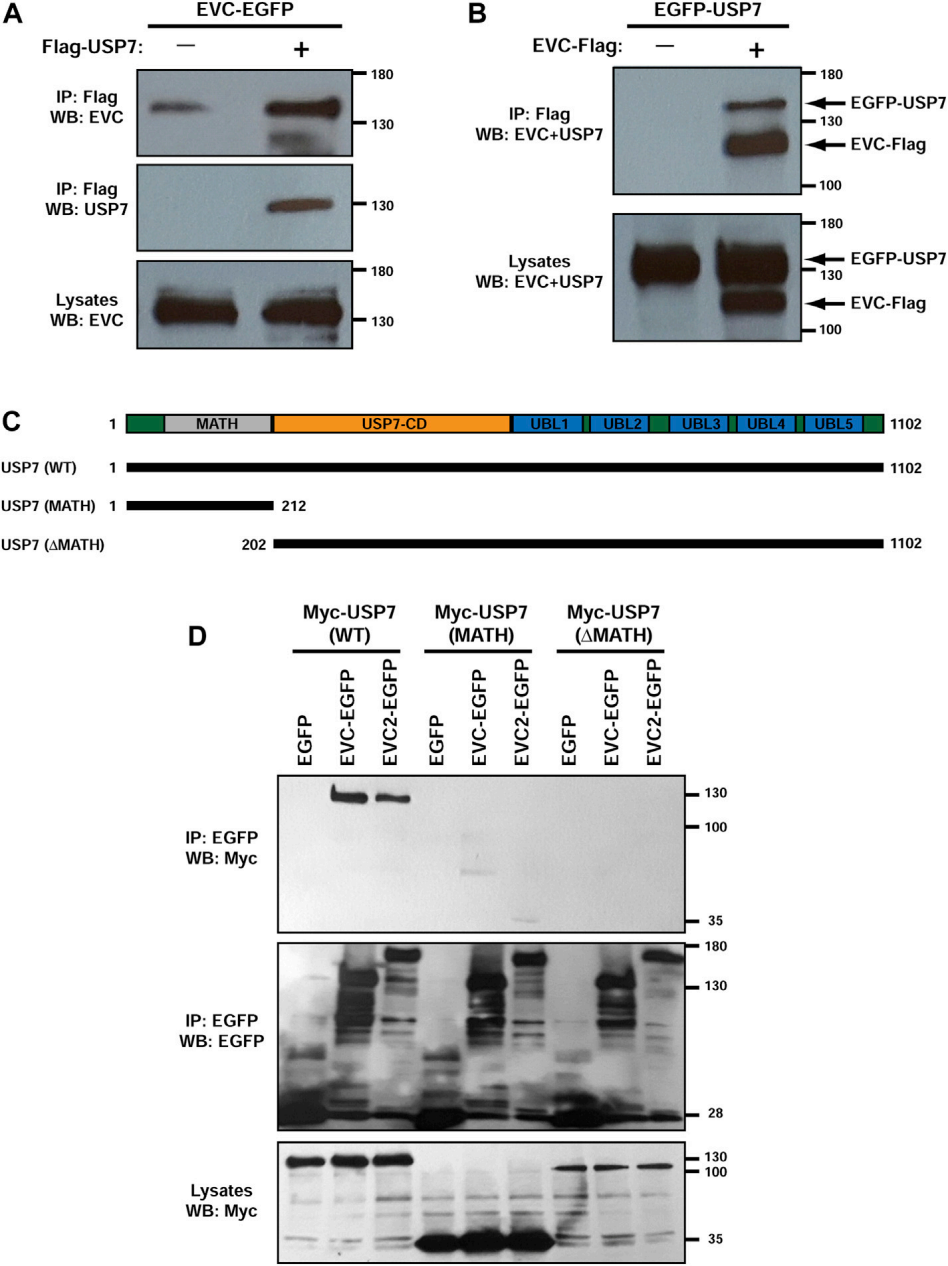
USP7 interacts with EVC-EVC2 in a MATH domain-dependent manner. **(A)** EVC-EGFP and Flag-USP7 were expressed in HEK293T cells as indicated. Lysates were immunoprecipitated (IP) with anti-Flag beads and analyzed by Western blot (WB) with the indicated antibodies. **(B)** Same anti-Flag immunoprecipitation (IP) experiment as in **(A)**, but using different constructs, as indicated. **(C)** Schematic of the USP7 protein with its central deubiquitinase catalytic domain (USP7-CD), its five C-terminal ubiquitin-like domains (UBL1-5), and its N-terminal Meprin and TRAF homology (MATH) domain. The lengths of the Myc-tagged USP7 constructs used in **(D)** are indicated below. **(D)** The indicated constructs were expressed in HEK293T cells as indicated. Lysates were immunoprecipitated (IP) with anti-EGFP beads and analyzed by Western blot (WB) with anti-EGFP and anti-Myc antibodies, as indicated. Molecular weight markers are shown on the right of all blots.

First, we sought to confirm the USP7-EVC interaction identified above. For this, we performed co-immunoprecipitation (co-IP) experiments in transfected HEK293T cells. Indeed, EVC-EGFP specifically associated with Flag-USP7 when the latter was immunoprecipitated with anti-Flag beads (Figure 2A). Likewise, EGFP-USP7 readily and specifically associated with EVC-Flag (Figure 2B), and so did Myc-USP7 with EVC-EGFP, and also with EVC2-EGFP (Figures 2C, D). These data confirm the interaction between USP7 and the EVC-EVC2 complex.

We also began probing which domains in USP7 are needed for these interactions. USP7 contains an N-terminal MATH/TRAF domain, followed by the catalytic domain, and five ubiquitin-like domains (UBLs) (Figure 2C) (Zapata et al., 2001; Bhattacharya et al., 2018). Deletion of the MATH domain in the Myc-USP7-ΔMATH construct appeared to abolish USP7’s interaction with both EVC-EGFP and EVC2-EGFP. However, the Myc-USP7-ΔMATH construct was expressed at lower levels than Myc-USP7-WT, so this might also be the reason why we observed no interaction. More conclusively, the MATH domain alone did not interact with EVC or EVC2 constructs, even though Myc-USP7-MATH was expressed more than WT (Figure 2D). Therefore, USP7’s MATH domain alone is not sufficient for interaction with EVC-EVC2, but it may be necessary for it. More detailed studies will be needed to clarify this.

**FIGURE 3:**
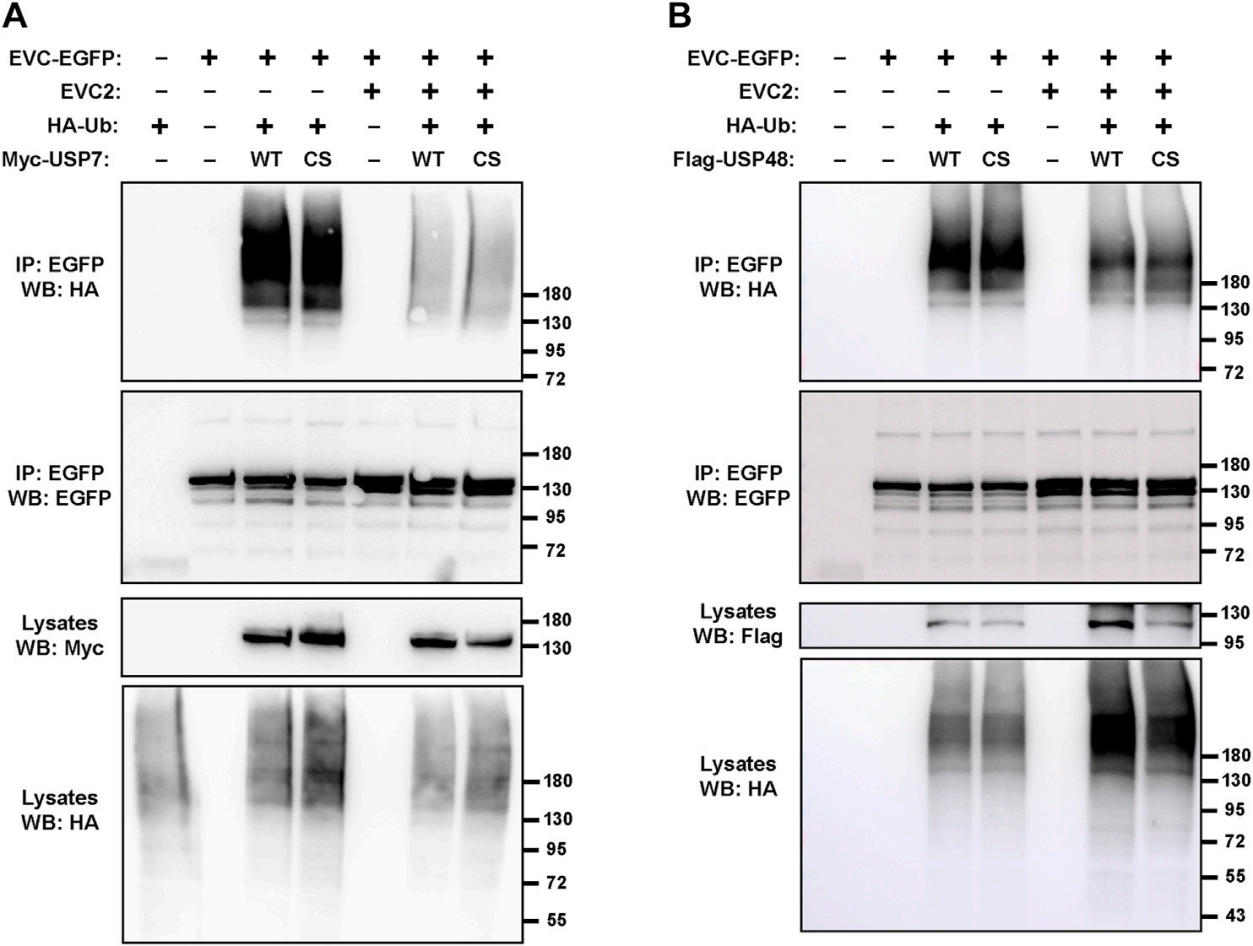
Deubiquitinating enzymes USP7 and USP48 do not affect EVC ubiquitination. **(A)** The indicated constructs were expressed in HEK293T cells, which were treated with 10 μM MG132 (proteasome inhibitor) the last 4 h before lysis. Minus signs indicate an equal amount of the corresponding empty vector was transfected instead (e.g., EGFP instead of EVC2-EGFP). Cell lysates were immunoprecipitated (IP) with anti-EGFP beads and analyzed by Western blot (WB) with the indicated antibodies. **(B)** Same experiment as in **(A)** but using Flag-USP48 instead of Myc-USP7. For both, WT is the wild type form, while CS is a catalytically inactive mutant. Molecular weight markers are shown on the right of all blots.

After confirming that USP7 and EVC-EVC2 interact, we explored the possible functional significance thereof. In particular, we checked whether EVC-EVC2 undergo ubiquitination, and if so, whether it is USP7-dependent. To test this, we carried out ubiquitination assays in HEK293T cells (Figure 3A). For this, we cotransfected these cells with HA-tagged ubiquitin, EVC-EGFP, and USP7 WT or C223S, a catalytically inactive USP7 mutant previously shown to function in a dominant negative manner (Sarkari et al., 2011; Zhou et al., 2015). We also treated these cells with MG132, which blocks proteasomal degradation of polyubiquitinated proteins (Lee and Goldberg, 1998). Under these conditions, we immunoprecipitated EVC-EGFP and looked at its ubiquitination by Western blot with anti-HA antibodies.

The results clearly indicated that EVC-EGFP undergoes ubiquitination (Figure 3A). Such ubiquitination includes a strong smear above 180 kDa, probably reflecting polyubiquitination, as well as two discrete bands near the predicted size of unmodified EVC-EGFP (140 kDa), which might reflect monoubiquitination at one or two sites in the protein. All these ubiquitinated bands were virtually identical when USP7-WT was transfected instead of USP7-C223S, indicating that this ubiquitination is independent of USP7 activity. In contrast, when untagged EVC2 was also transfected, all ubiquitinated bands were strongly reduced (Figure 3A). Altogether, these data indicate that EVC proteins undergo ubiquitination, which is unaffected by USP7 but strongly depends on whether EVC-EVC2 find each other to form their heterodimeric and mutually stabilizing complex.

Besides USP7, we also tested the effect on EVC ubiquitination of USP48, another deubiquitinating enzyme known to regulate Hh signaling (Zhou et al., 2017; Sanchez-Bellver et al., 2022). As with USP7, transfection with USP48 (WT or the C98S dominant negative) did not make any difference in the observed EVC-associated ubiquitination (Figure 3B). In presence of USP48, we also observed the protective effect of EVC2 on EVC ubiquitination, if the amount of HA-ubiquitin in the lysates is taken into account (Figure 3B). Hence, we observed no effects of USP7 or USP48 in these experiments, so the enzymes controlling EVC ubiquitination remain unknown.

Since USP7 interacts with GLI transcription factors, we also considered whether EVC proteins might affect this interaction (Zhou et al., 2015). To test this, we first used CRISPR-Cas9 technology to generate *EVC*-knockout (KO) HEK293T cells (Supplementary Figure S1A). We then performed USP7-GLI1 co-IP experiments comparing *EVC* WT and KO cells. In these experiments, Flag-GLI1 robustly co-immunoprecipitated with EGFP-USP7 regardless of EVC presence (Supplementary Figure S1B). Overexpression of EVC-EVC2 in wild type HEK293T also had no effect on this interaction (Supplementary Figure S1C).

### 2.3 Monoubiquitination of EVC-EVC2 cytosolic tails targets them for degradation

**FIGURE 4:**
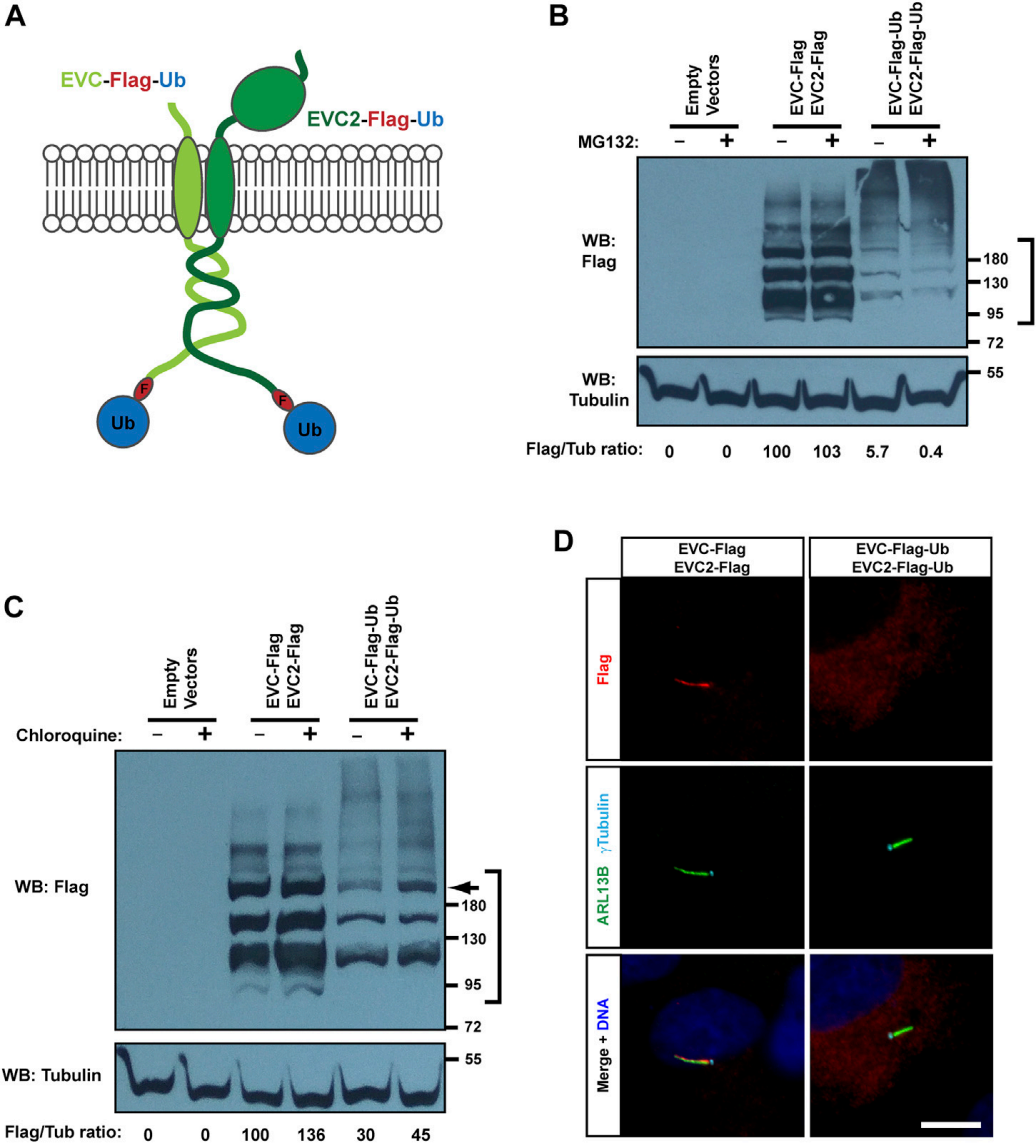
Monoubiquitination of EVC-EVC2 reduces their protein levels and ciliary targeting. **(A)** Diagram of the EVC and EVC2 constructs used in this figure. The full length EVC and EVC2 proteins (light and dark green, respectively) are fused in their intracellular C-termini to a Flag epitope (red) followed by ubiquitin (blue). **(B)** The fusion proteins from **(A)**, and the indicated controls, were expressed in HEK293T cells, which were treated with 10 μM MG132 (proteasome inhibitor) the last 4 h before lysis. Cell lysates were analyzed by Western blot (WB) with Flag and Tubulin antibodies, as indicated. The region marked on the right was used to quantify Flag signal intensity in each sample, which is shown below as a Flag/Tubulin ratio, and as percentage relative to the third lane. Data comes from one independent experiment. Molecular weight markers are on the right. **(C)** Same as in **(B)**, but treating with 10 μM chloroquine for 24 h (lysosome inhibitor), instead of MG132. Arrow points to band of ubiquitinated EVC’s, whose levels increase 2.3x in presence of chloroquine. **(D)** hTERT-RPE1 cells were transfected with the indicated constructs, starved to induce ciliation, fixed, and stained with antibodies against Flag (red), ciliary marker ARL13B (green), basal body marker γ-tubulin (cyan), and with DAPI for DNA (blue). Scale bar, 10 μm.

We next explored what happens if the C-terminal cytosolic tails of EVC and EVC2 are monoubiquitinated. To do this, we followed a common approach, namely, fusing monoubiquitin to the C-terminal end of these proteins (Figure 4A) (Haglund et al., 2003; Husnjak and Dikic, 2012). We then looked at whether this affects protein levels by Western blot of transfected HEK293T cell lysates. Compared to the co-transfected control constructs (EVC-Flag + EVC2-Flag), the protein levels of the monoubiquitinated constructs (EVC-Flag-Ub + EVC2-Flag-Ub) were strongly reduced, coupled to an increase of high molecular weight bands (Figure 4B). Addition of MG132 did not increase any of the observed bands, as expected, given that the proteasome does not recognize monoubiquitin, requiring for its recognition K48-linked tetraubiquitin or longer chains (Figure 4B) (Ding et al., 2019).

Instead, monoubiquitination of transmembrane proteins often affects their trafficking and lysosomal degradation (Husnjak and Dikic, 2012). To test this, we used chloroquine to interfere with lysosomal function (Mauthe et al., 2018). In this case, we did observe a clear increase in the protein levels of monoubiquitinated EVC-EVC2, especially of a high molecular weight band (≈200 kDa) that seems to correspond to a modified, perhaps glycosylated, form of EVC or EVC2 (Figure 4C). As above, a smear of unknown significance was observed at very high molecular weights (>200 kDa). This smear was again stronger for the monoubiquitinated constructs, and appeared to mildly increase with chloroquine (Figure 4C).

We then tested, by immunofluorescence staining, whether chloroquine affects endogenous EVC in MEFs (Supplementary Figure S2). However, there was no obvious EVC accumulation at the EvC zone or elsewhere after 16 h of chloroquine treatment, suggesting that EVC lysosomal turnover is slow under normal conditions.

We then examined the effect of monoubiquitin on EVC-EVC2 subcellular localization in transfected hTERT-RPE1 cells. Consistent with the Western blot data, monoubiquitinated EVC-EVC2 showed weak staining, and virtually no ciliary accumulation, as opposed to controls (Figure 4D). Therefore, we conclude that monoubiquitination of EVC-EVC2 reduces their protein levels throughout the cell and in cilia.

In hTERT-RPE1, the co-transfected control proteins (EVC-Flag + EVC2-Flag) always accumulated in cilia, but the staining was not always confined to the EvC zone, sometimes spanning the whole cilium (as in the example in Figure 4D). This may be due to saturation of EvC zone-binding sites, caused by EVC-EVC2 overexpression. Still, plenty of transfected cells also displayed specific EvC zone localization, which we confirmed by co-staining the EVC proteins with transition zone markers, and by plotting signal intensities along cilia (Supplementary Figure S3). In the same manner, we also confirmed EvC zone localization in IMCD3 cells, which we also use below to study EVC protein targeting (Supplementary Figure S4). Instead, in HEK293T cells, known for their high expression levels of exogenous proteins, overexpressed EVC-EVC2 localized uniformly in cilia (in the small proportion of cells that formed them) (Supplementary Figure S5).

### 2.4 The EVC-EVC2 complex undergoes sumoylation

Since EVC-EVC2 can be ubiquitinated, we wondered if they can also be modified by ubiquitin-like proteins, such as the small ubiquitin-like modifier (SUMO) proteins (Vertegaal, 2022). This possibility was further suggested by *in silico* analyses of EVC and EVC2’s cytosolic tails, which contain putative sumoylation motifs (Kumar et al., 2020). Additionally, localization of some ciliary proteins, like Smoothened and ADCY3, is regulated by modification with SUMO2/3 (Li et al., 2012; McIntyre et al., 2015; Ma et al., 2016).

**FIGURE 5:**
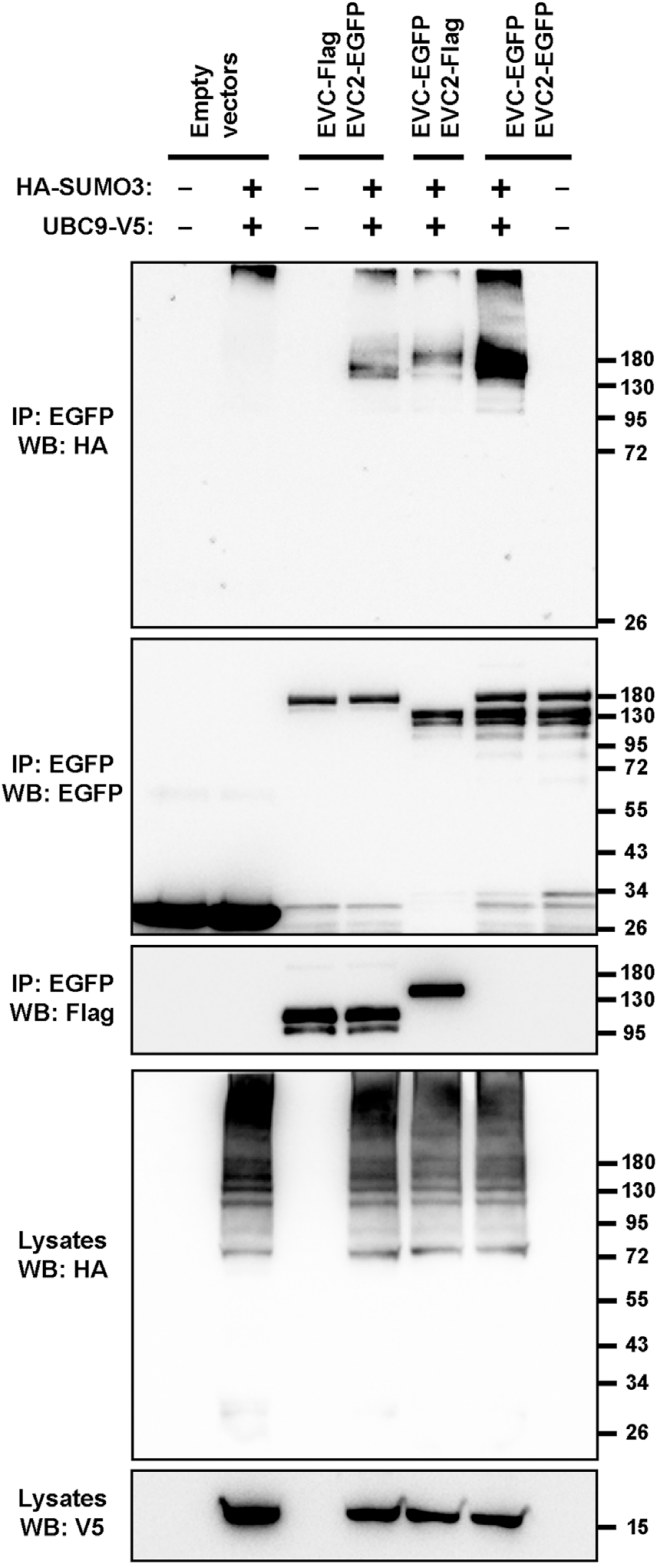
The EVC-EVC2 complex can undergo sumoylation. The indicated proteins, including Flag or EGFP-tagged EVC and EVC2, the V5-tagged SUMO-conjugating enzyme UBC9, and HA-tagged SUMO3 were expressed in HEK293T cells. Cell lysates were immunoprecipitated (IP) with anti-EGFP beads and analyzed by Western blot (WB) with the indicated antibodies. Molecular weight markers are on the right.

We addressed this by first performing sumoylation assays in transfected HEK293T cells. For this, tagged EVC and EVC2 constructs were co-expressed with HA-SUMO3 and the SUMO-conjugating enzyme UBC9 (Vertegaal, 2022). Under these conditions, aside from a non-specific band at the very top of the blot, clear and specific SUMO3 conjugation was observed in the EVC-EVC2 immunoprecipitates (Figure 5). Thus, EVC proteins can indeed undergo sumoylation.

### 2.5 Sumoylation of EVC-EVC2 cytosolic tails enhances their ciliary EvC zone localization

**FIGURE 6:**
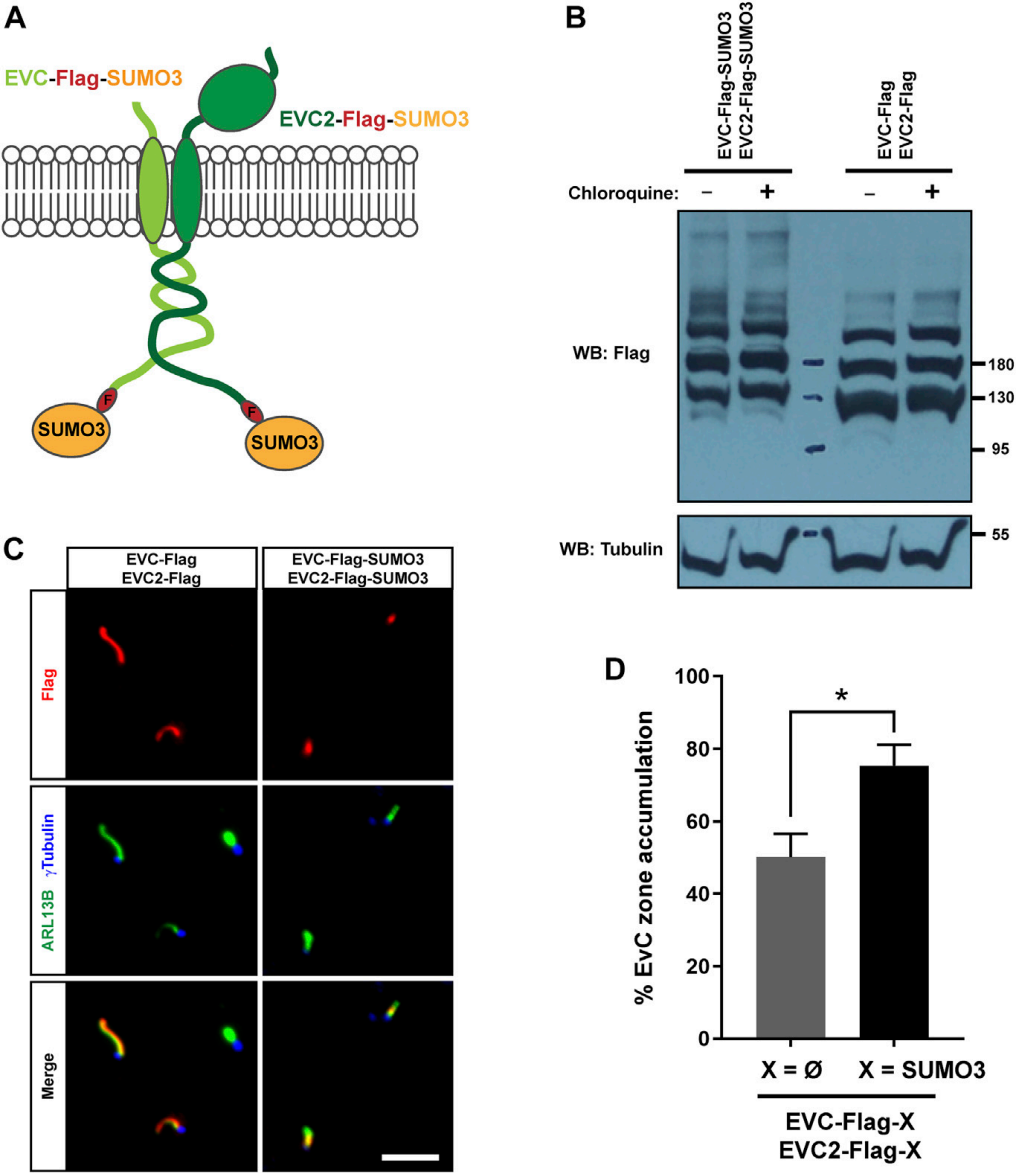
EVC-EVC2 complex sumoylation enhances its EvC zone targeting. **(A)** Diagram of the EVC and EVC2 constructs used in this figure. The full length EVC and EVC2 proteins (light and dark green, respectively) are fused in their intracellular C-termini to a Flag epitope (red) followed by SUMO3 (orange). **(B)** The fusion proteins from **(A)**, or the indicated controls, were expressed in HEK293T cells, which were treated with 10 μM chloroquine for the last 24 h before cell lysis. Cell lysates were analyzed by Western blot (WB) with the indicated antibodies. Molecular weight markers on the right. These samples were run in parallel to those in Figure 4C. **(C)** IMCD3 cells were transfected with the indicated constructs and processed for immunofluorescence with the indicated antibodies. Scale bar, 5 μm. **(D)** Quantitation of data from **(C)**. Percent of transfected cell cilia showing anti-Flag staining concentrated at the EvC zone, as opposed to evenly distributed along the cilium. Data are mean ± SEM of *n* = 3 independent experiments. Asterisk denotes *p* <0.05 in unpaired Student’s t-test.

To test a possible role for sumoylation in EVC-EVC2 protein levels and/or targeting, we created EVC-Flag-SUMO3 and EVC2-Flag-SUMO3 constructs analogous to the monoubiquitin ones used above (Figure 6A). When we analyzed these constructs by Western blot in HEK293T, we did not observe any strong differences in their protein levels compared to controls, and this did not change when adding chloroquine (Figure 6B). Thus, unlike monoubiquitination, sumoylation of EVC-EVC2 cytosolic tails has no strong effects on protein levels.

Accordingly, EVC/EVC2-Flag-SUMO3 fusions were readily visible inside cilia by immunofluorescence in transfected IMCD3 cells (Figure 6C). However, while EVC/EVC2-Flag controls were often seen all along the cilium under these conditions, the SUMO3 fusions were more consistently seen at the EvC zone. Quantitation of the frequency of EvC zone accumulation indeed showed a significant increase in the SUMO3 condition (Figure 6D). This suggests that SUMO3 conjugation to EVC-EVC2 cytosolic tails enhances their localization to the EvC zone.

To see if this effect is important for EVC localization under basal conditions, we checked the effect of ginkgolic acid (GA), a sumoylation inhibitor, on endogenous EVC localization in MEFs (Fukuda et al., 2009). We first treated cells with 10 μM GA for 24 h, which led to massive cell death. Cells did not die when treated with 5 μM GA for 24 h, and this did not perturb EVC accumulation at the EvC zone. However, immunoblotting showed no effect of GA on SUMO2/3 levels either, indicating that the 5 µM dose was insufficient for our purposes (data not shown).

We also checked whether SUMO2/3 or ubiquitin was specifically detected in immunoprecipitates of endogenous EVC in serum-starved MEFs treated or not with MG132 or chloroquine. No endogenous ubiquitination or sumoylation was seen in this experiment, suggesting the levels of these modifications are very low, or our detection method not powerful enough to detect them (Supplementary Figure S6). Thus, the role of sumoylation and ubiquitination on endogenous EVC regulation remains an open question.

### 2.6 Sumoylation of EVC-EVC2 cytosolic tails enhances their interaction with EFCAB7

**FIGURE 7:**
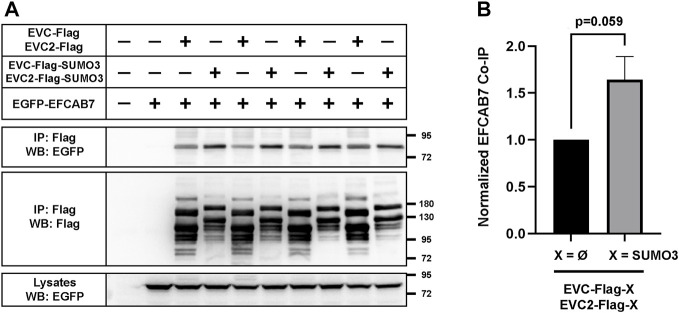
EVC-EVC2 complex sumoylation enhances its interaction with EFCAB7. **(A)** The indicated constructs were expressed in HEK293T cells. Cell lysates were immunoprecipitated (IP) with anti-Flag beads and analyzed by Western blot (WB) with the indicated antibodies. Molecular weight markers on the right. Negative signs indicate empty vector was transfected instead: pEGFP or pFlag. The lane pairs 3-4, 5-6, 7-8 and 9-10 are technical replicates, except for variations in the amount of transfected constructs, as follows: i) 3.3 µg of each of the three plasmids in lanes 1–4; ii) 1.65 µg of each in lanes 9-10; iii) 1.65 µg of EFCAB7 plasmid and 3.3 µg of the rest in lanes 5-6; iv) 1.65 µg of each EVC/EVC2 plasmid and 3.3 µg of EFCAB7 in lanes 7-8. All these amounts were transfected in 10 cm culture dishes. **(B)** Quantitation of EFCAB7 co-immunoprecipitation (co-IP) by the EVC-EVC2 complex from *n* = 3 independent experiments, including the one in **(A)**. The band intensities of EGFP-EFCAB7 in the IPs were normalized to the intensities of both i) EVC/EVC2-Flag, with or without SUMO3, in IPs, and ii) EFCAB7 in lysates. In each experiment, the X = Ø condition was additionally normalized to 1. Data are mean ± SEM (*n* = 3). The *p*-value reflects the outcome of a two-tailed, unpaired, homoscedastic Student’s t-test.

EvC zone targeting of the EVC-EVC2 complex is mediated by its interaction with the EFCAB7-IQCE complex, and more specifically by the interaction between EFCAB7’s ECH2 domain with the Weyers peptide at EVC2’s C-terminus (Pusapati et al., 2014). Hence, it would make perfect sense if the enhanced EvC zone targeting induced by SUMO3 was mediated by increased association to EFCAB7. To test this, we again performed co-IP experiments in HEK293T. In these experiments, SUMO3 fusion to the cytosolic tails of EVC-EVC2 consistently boosted the amount of EGFP-EFCAB7 pulled down by the EVC-EVC2 complex (Figure 7A). Quantitation of these data across three independent experiments showed a very strong trend (*p* = 0.06) (Figure 7B). Thus, it seems likely that SUMO3 promotes EvC zone targeting by reinforcing the interaction between the EVC-EVC2 complex and EFCAB7.

### 2.7 EvC zone targeting of EVC-EVC2 requires two separate motifs in EVC2’s W-peptide

As mentioned above, the W-peptide, including the last 43 aa at EVC2’s C-terminus (aa 1178-1220 in mouse EVC2), is critical for EvC zone accumulation of the EVC-EVC2 complex, as opposed to a more homogeneous distribution along the entire ciliary length (Dorn et al., 2012; Caparros-Martin et al., 2013; Pusapati et al., 2014). Within the W-peptide, the FV motif (aa 1185-86) was shown to be essential (Dorn et al., 2012). Nevertheless, this motif does not appear to act alone, since deleting the last 24 aa in the W-peptide equally perturbs EvC zone targeting, even though this leaves the FV motif intact (Caparros-Martin et al., 2013).

**FIGURE 8:**
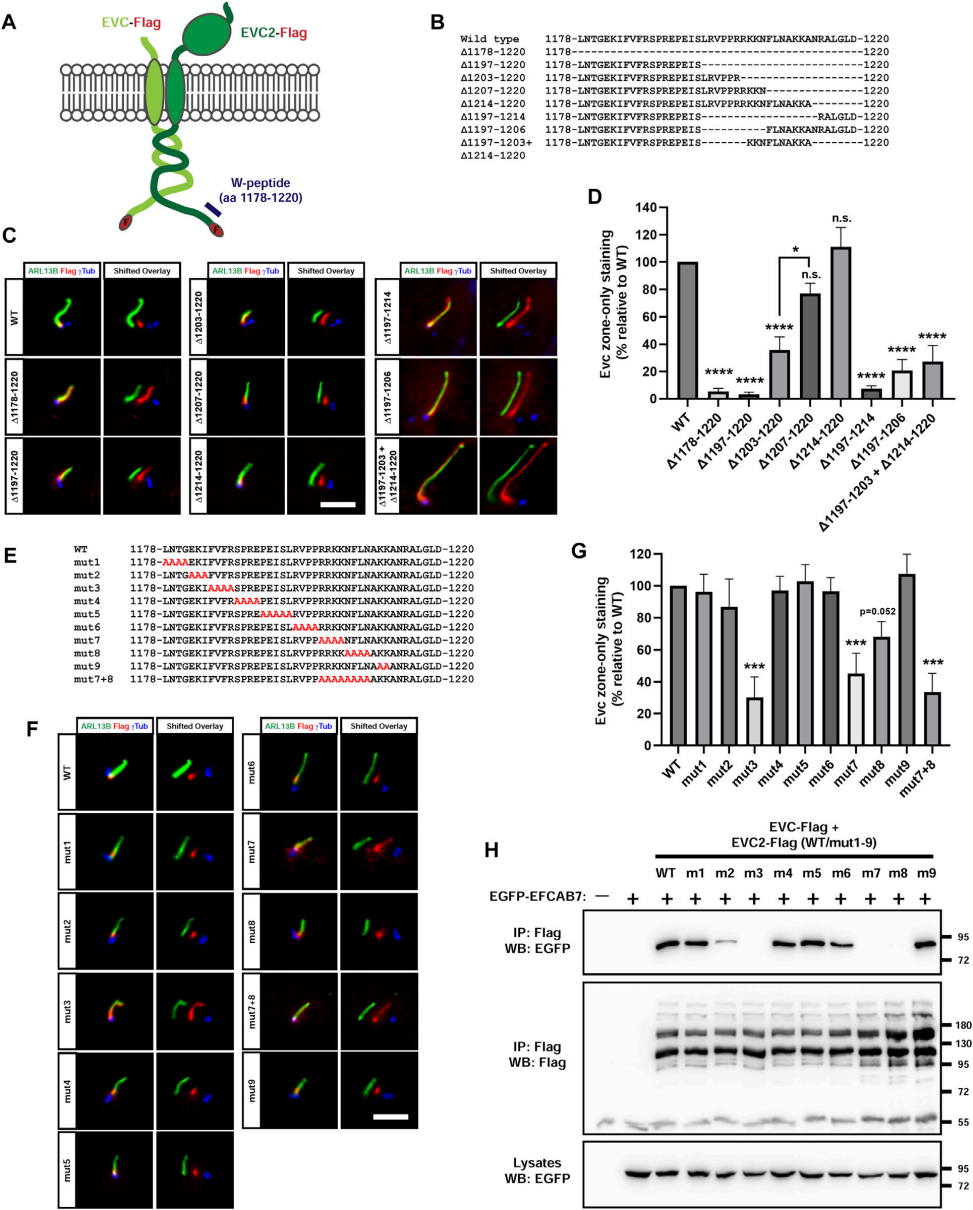
EFCAB7 binding and EvC zone targeting of the EVC-EVC2 complex requires two separate motifs in EVC2’s Weyers peptide. **(A)** Diagram of the Flag-tagged EVC-EVC2 constructs used in this figure to mutagenize the Weyers peptide (W-peptide), spanning the last 43 aa of EVC2. **(B)** W-peptide sequence in wild type EVC2 and in the indicated deletion mutants. **(C)** IMCD3 cells transfected with the indicated constructs were analyzed by immunofluorescence. In the right panels (shifted overlays), the green and blue channels have been horizontally shifted to the left and right, respectively, relative to the red channel, to separately visualize the individual signals. The aligned merge is shown on the left panels. Scale bar, 5 μm. **(D)** Quantitation of the experiments in **(C)**. The *y*-axis shows the percentage of EvC zone-only localization for each construct relative to WT, which was normalized to 100%. Data are mean ± SEM of *n* = 7,3,2,3,3,3,3,3,4 (left to right) independent experiments. Data were analyzed by one-way ANOVA, followed by Tukey’s multiple comparisons tests, whose *p*-values are indicated as: *p* <0.0001 (****), *p* <0.05 (*), or not significant (n.s.). Significance is relative to WT unless otherwise indicated. **(E)** W-peptide sequence in wild type EVC2 and in the indicated alanine substitution mutants. **(F)** The mutants from **(E)** were analyzed as in **(C)**. **(G)** Quantitation of the experiments in **(F)** was performed as in **(D)**. Data are mean ± SEM of *n* = 9,3,3,3,3,3,3,6,6,3,3 (left to right) independent experiments and were analyzed by one-way ANOVA followed by Dunnet tests comparing each sample to WT. Three asterisks indicate *p* <0.001. **(H)** The indicated proteins were expressed in HEK293T cells (m1-m9: mut1-mut9). Cell lysates were immunoprecipitated (IP) with anti-Flag beads and analyzed by Western blot (WB) with the indicated antibodies. Molecular weight markers are on the right.

To clarify this issue, we generated a battery of deletion mutants in EVC2’s W-peptide (Figures 8A, B). First, we confirmed that deleting the entire W-peptide’s 43 aa (Δ1178-1220), or its last 24 aa (Δ1197-1220), abolishes the EVC-EVC2 complex’s tendency to specifically accumulate at the EvC zone (Figures 8C, D). Such accumulation was still strongly reduced by deletion of the last 18 aa (Δ1203-1220), but was normal when only the last 14 aa (Δ1207-1220) or 7 aa (Δ1214-1220) were deleted. Thus, EVC2 residues 1197–1206 harbor an additional EvC zone-targeting motif. This was confirmed by the mistargeting of Δ1197-1206 and Δ1197-1214. Finally, the double Δ1197-1203+Δ1214-1220 deletion showed that essential residues are present within 1197–1203 (Figures 8C, D).

We also performed alanine substitution mutagenesis of the entire W-peptide, except for the last 7 residues, which we already knew to be dispensable for EvC zone targeting (Figure 8E). As expected, removal of the FV motif in the mut3 mutant (FVFR > AAAA in aa 1185-1188), abolished EvC zone targeting (Figures 8F, G). The same was true for mut7 (RRKK > AAAA in aa 1202-1205), and to a lesser extent for mut8 (NFLN > AAAA in aa 1206-1209) (Figures 8F, G). Combining mut7 and mut8 did not further impair targeting (Figures 8F, G). No effect whatsoever was observed with mut1, 2, 4, 5, 6 and 9. Altogether, our deletion and substitution data indicate that EVC2’s W-peptide contains two essential motifs for EvC zone targeting: the FV motif (aa 1185-1186) and the RRKKN motif (aa 1202-1206), a model which is fully consistent with previous work (Dorn et al., 2012; Caparros-Martin et al., 2013).

Since the FV motif promotes EvC zone targeting by allowing EVC2’s W-peptide interaction with EFCAB7, we tested whether the same was true for the RRKKN motif. Indeed, of all the alanine mutants (mut1-9), none affected EFCAB7 binding except mut3, mut7 and mut8, all of which completely abolished the interaction (Figure 8H). Therefore, both FV and RRKKN motifs are required for EFCAB7 binding, thus explaining their requirement for EvC zone targeting.

## 3 Discussion

In this work, we have generated an interactomic dataset for the endogenous EVC protein in mouse fibroblasts (Figure 1). Several facts point to the quality of this resource and its potential usefulness to the scientific community: i) physiological relevance: endogenous EVC was used as bait in a ciliated and Hh-responsive cell type; ii) specificity: *Evc*-null cells were used as negative control; iii) reproducibility: three independent experiments; iv) validation: EVC’s known interactors EVC2, EFCAB7 and IQCE were all among the top hits; v) novelty: many new putative interactors were identified. Since we have not explored most of these hits further, the field is wide open for cell biologists to generate and test hypotheses based on these data. To facilitate this, all raw data, and our analyses thereof, are available in Supplementary Data File 1.

The EVC interactome list displayed in Figure 1C was generated using fairly stringent criteria (described in Supplementary Data File 1), in order to minimize false positives. This, however, likely led to the exclusion of some *bona fide* interactors. Hence, future studies should not only focus on the shortlisted hits, but also on the rest of the data. Still, the ten novel interactors in Figure 1C seem the most promising and already provide plenty of hypotheses to test, such as possible roles for EVC-EVC2 in: i) tight junction assembly or function, based on interactors CGN and MPP7 (Bohl et al., 2007; Stucke et al., 2007; Van Itallie and Anderson, 2014); ii) extracellular matrix biology, based on LOXL2 and HS2ST1 (Teixeira et al., 2020; Liburkin-Dan et al., 2022); iii) membrane permeability control, based on UPK1B-UPK3B (Wu et al., 2009); iv) cell signaling and stress responses, based on MSLN and ANKZF1 (Koyama et al., 2017; van Haaften-Visser et al., 2017; Kuroha et al., 2018; Verma et al., 2018); or v) protein phosphorylation and deubiquitination, based on PRKACA and USP7 (Bangs and Anderson, 2017; Bhattacharya et al., 2018). In summary, this EVC interactome is a potentially rich resource, if enough effort is devoted to mine it, including further validation of the interactions and assessment of their biological meanings.

The hit that most caught our attention initially was USP7, given its connection to Hh signaling and deubiquitination. USP7 is an essential protein cells cannot live long without, so knockdown approaches to study its function are challenging. Among USP7’s many important targets are p53, PTEN, β-catenin, PLK1, and GLI transcription factors, to name a few (Zhou et al., 2015; Bhattacharya et al., 2018). After confirming that USP7 and EVC-EVC2 do indeed interact (Figure 2), we failed to detect any deubiquitinating activity of USP7 on the EVC-EVC2 complex, and the same was true for USP48, also involved in Hh signaling (Figure 3) (Zhou et al., 2017; Sanchez-Bellver et al., 2022). We also failed to see any effect of EVC knockout or overexpression on the USP7-GLI1 interaction (Supplementary Figure S1). Thus, we have not found any functional connections between USP7 and EVC-EVC2. Therefore, whether such connections exist remains an open question.

In any case, our assays with USP7 clearly showed that EVC-EVC2 undergo ubiquitination, which strongly increases when the complex is not allowed to form (Figure 3). The observed ubiquitination includes a high molecular weight smear, a well-known hallmark of polyubiquitination, which is typically linked to proteasome degradation. Thus, we hypothesize that, as occurs with other protein complexes, EVC-EVC2 complex assembly protects these proteins from ubiquitin-dependent proteasomal degradation (Husnjak and Dikic, 2012).

In addition to polyubiquitination, we also saw a couple of discrete bands at sizes consistent with EVC monoubiquitination at one or two sites. Since the proteasome only recognizes tetraubiquitin chains or longer, monoubiquitination cannot mediate proteasomal degradation, instead typically mediating endosomal and/or lysosomal targeting of transmembrane proteins (Husnjak and Dikic, 2012; Ding et al., 2019). Since this was a plausible hypothesis for EVC-EVC2, we tested it by adding monoubiquitin to both their C-termini (Figure 4). This leads to a strong reduction in EVC-EVC2 protein levels. Such reduction is not rescued with a proteasome inhibitor, but is partially rescued by a lysosome inhibitor, chloroquine, consistent with our hypothesis.

The fact that chloroquine only raises levels of the higher molecular weight EVC-EVC2 bands (especially one at ≈200 kDa) can potentially be explained if those higher bands correspond to mature forms of EVC-EVC2 (forms that are, presumably, fully glycosylated, have reached the plasma membrane, and are therefore more susceptible to endolysosomal trafficking). On the other hand, the lower chloroquine-unaffected bands may correspond to immature EVC-EVC2 forms that are still in ER-Golgi and are less exposed to endolysomal trafficking machinery. However, these hypotheses remain speculative. Likewise, we can only speculate about the nature of the smear specifically seen with the monoubiquitinated constructs (Figures 4B, C). This smear is unaffected by MG132, so it does not appear to reflect K48-linked polyubiquitination of these constructs. Another hypothesis is that the monoubiquitin moiety increases EVC/EVC2 glycosylation as they passage through ER-Golgi. This could also explain why this smear increases with chloroquine, but more experiments are needed to clarify these points.

Given that sumoylation controls targeting of some ciliary transmembrane proteins, like Smoothened and adenylate cyclase 3, we also tested how sumoylation affects EVC-EVC2 (Li et al., 2012; McIntyre et al., 2015; Ma et al., 2016). There are three different SUMO proteins (SUMO1-2-3), all of which share homology with ubiquitin and are conjugated to lysine residues of target proteins in essentially the same way. Since SUMO1 modification typically occurs in the nucleus, and since SUMO2-3 are structurally and functionally almost identical, and both function in the cytoplasm, we decided to look at how SUMO3 affects EVC-EVC2 (Vertegaal, 2022).

After finding that EVC-EVC2 can indeed undergo conjugation to SUMO3 in cell-based assays (Figure 5), we found that SUMO3 modification of EVC and EVC2’s cytosolic tails enhances EvC zone targeting of the complex, and does so by promoting its interaction with EFCAB7 (Figures 6, 7). This is perfectly consistent with EFCAB7’s previously described role as docking site for EVC-EVC2 in the EvC zone (Pusapati et al., 2014). Thus, our data point to sumoylation as a novel mechanism controlling EvC zone targeting of the EVC-EVC2 complex. Nevertheless, whether and how this mechanism is used by the endogenous EVC proteins, rather than the overexpressed SUMO3 fusions we used, is an important question that remains to be addressed, as is the case for monoubiquitination. Our initial attempts at detecting endogenous sumoylated or ubiquitinated forms of the EVC proteins have been unsuccessful (Supplementary Figure S6).

Lastly, we have identified the RRKKN motif in mouse EVC2’s W-peptide as the missing motif required for EvC zone targeting of the EVC-EVC2 complex (Figure 8). Thus, such targeting requires both the FV motif (aa 1185-6, previously reported by Dorn et al. and confirmed herein), and the RRKKN motif (aa 1202-6). The requirement for the RRKKN motif explains why some W-peptide deletions not affecting the FV motif also disrupt EvC zone targeting (Caparros-Martin et al., 2013). All other residues within the W-peptide are dispensable. These motifs are highly conserved in human EVC2, where FV and RRKKN appear as IV (aa 1257-8) and RKKKN (aa 1290-4), respectively. Thus, our findings now allow us to predict that mutations disrupting aa 1257-8 and/or 1290-94 in human EVC2 will cause WAD, a prediction that is consistent with currently available data (D’Asdia et al., 2013; Ruiz-Perez and Goodship, 2009). Interestingly, a duplication of aa 1293-1300 in human EVC2 was found in association with Meckel-Gruber syndrome (MKS), a severe ciliopathy (Shaheen et al., 2013). However, this duplication leaves the RKKKN motif intact, duplicating only its last two residues, so we do not expect it to disrupt EFCAB7 binding, as occurs with W-peptide deletions.

In summary, here we have identified: i) novel EVC interactors to guide future functional explorations; ii) ubiquitination and sumoylation as posttranslational modifications regulating EVC-EVC2 protein levels and ciliary EvC zone targeting; and iii) the RRKKN motif as an essential component of the WAD ciliopathy-deleted EVC2’s W-peptide.

## 4 Materials and methods

### 4.1 Reagents and antibodies

The monoclonal mouse anti-EVC antibody used for the interactomics experiments has been described elsewhere (Pacheco et al., 2012). Mouse monoclonal antibodies against acetylated alpha-tubulin (Sigma, T7451), alpha-tubulin (Proteintech, 66031-1-Ig), gamma-tubulin (Santa Cruz, sc-17787), and EGFP (Proteintech, 66002-1-Ig) were used as described previously (Cilleros-Rodriguez et al., 2022), as was the case for rabbit polyclonal antibodies against Myc epitope (Proteintech, 16286-1-AP) and EGFP (Proteintech, 50430-2-AP), as well as for the Chromotek GFP-Trap_MA beads (Proteintech, gtma), and for all secondary antibodies. Other antibodies used here include: mouse anti-Flag (Sigma, F1804, IF: 1:200, WB: 1:2000), mouse anti-Flag (Proteintech, 66008-3-Ig, IF: 1:200, WB: 1:2000), mouse anti-ARL13B (Proteintech, 66739-1-Ig, IF: 1:100), mouse anti-V5 (Thermofisher, MA5-15253, WB: 1:1000), rabbit anti-human EVC (Sigma, HPA016046, WB: 1:1000), rabbit anti-USP7 (Santa Cruz, sc-30164, WB: 1:500), rabbit anti-AHI1 (Proteintech, 22045-1-AP, IF: 1:50), rabbit anti-MKS1 (Proteintech, 16206-1-AP, IF: 1:50), rabbit anti-ubiquitin (Proteintech, 10201-2-AP, WB: 1:1000), rabbit anti-SUMO2/3 (Proteintech, 11251-1-AP, WB: 1:1000), and rat anti-HA (Proteintech, 7c9, WB: 1:1000). Other reagents include mouse anti-Flag M2 magnetic beads (Sigma, M8823), SAG (Cayman, #11914), MG132 (Alfa Aesar, J63250), and chloroquine (Acros Organics, #45524).

### 4.2 Cell lines and transfections

*Evc* ^+/+^ and *Evc* ^*−*/−^ mice and MEFs (immortalized by retroviral delivery of SV40 large and small T antigens) were described previously (Ruiz-Perez et al., 2007; Blair et al., 2011; Pacheco et al., 2012; Caparros-Martin et al., 2013; Piceci-Sparascio et al., 2020). All cell lines were incubated at 37°C in a humidified atmosphere with 5% CO_2_, and were regularly tested to confirm they were mycoplasma-free. All cell lines were kept in basal medium (DMEM for MEFs and HEK293T; DMEM/F12 for hTERT-RPE1 and IMCD3), supplemented with 10% fetal bovine serum (FBS). For passaging, TrypLE was used (Thermofisher). HEK293T were transfected using the calcium phosphate method and lysed 40–48 h later. IMCD3 and hTERT-RPE1 cells were reverse transfected using JetPrime (Polyplus-transfection) and fixed 48 h later for cilia analysis, as reported previously (Cilleros-Rodriguez et al., 2022). Generation of *EVC*-null HEK293T cell lines by CRISPR was performed as previously described (Barbeito et al., 2021; Cilleros-Rodriguez et al., 2022), using the plasmids described below.

### 4.3 Plasmids and mutagenesis

pCAN-myc-USP7 WT and C223S plasmids were gifts from Dr. Lori Frappier (Sarkari et al., 2011), whereas pcDNA3-myc-USP7 WT, MATH (1-212) and ΔMATH (Δ202-1102) were gifts from Dr. Juan M. Zapata (Zapata et al., 2001). *USP7* WT coding sequence (CDS) was subcloned into pEGFP-C1 to make pEGFP-USP7. Mouse *Usp7* CDS was amplified by RT-PCR from MEFs to create pFlagCMV4-MmUSP7. For pEGFP-EFCAB7, mouse *Efcab7* CDS (AAI12328) was obtained by RT-PCR from MEFs and cloned into pEGFP-C1. pMT123-HA-Ub was a gift from Dr. Dirk Bohmann (Treier et al., 1994). HA-SUMO3 and UBC9-V5-expressing plasmids were gifts from Dr. Ronald T. Hay (Tatham et al., 2001; Girdwood et al., 2003; Fernandez-Lloris et al., 2006), and pcDNA3-Flag-USP48 (WT and C98S) were gifts from Dr. George Mosialos (Tzimas et al., 2006). The Flag-GLI1 plasmid was obtained by inserting mouse *Gli1* CDS into pFlagCMV4. Plasmids encoding untagged full length mouse EVC and EVC2 have been reported elsewhere (Caparros-Martin et al., 2013). Full length mouse *Evc* and *Evc2* CDS were cloned into pEGFP-N1 to create EVC-EGFP and EVC2-EGFP, from which we obtained EVC-Flag and EVC2-Flag plasmids, by replacing EGFP with Flag. To do this, we excised EGFP using AgeI-NotI digestion. The resulting open plasmid was then ligated with annealed primers encoding the Flag epitope followed by a stop codon, and flanked by AgeI-NotI cohesive ends. To generate the EVC-Flag-Ub, EVC2-Flag-Ub, EVC-Flag-SUMO3 and EVC2-Flag-SUMO3 plasmids, the CDSs of ubiquitin (Ub) and SUMO3 were PCR-amplified with primers adding AgeI targets on both sides, a Flag epitope at the N-terminus, and a stop codon at the end. The resulting AgeI-Flag-(Ub/SUMO3)-stop-AgeI amplicons were then inserted into the AgeI site of EVC-EGFP and EVC2-EGFP. The resulting plasmids, therefore, express (EVC/EVC2)-Flag-(Ub/SUMO3), but not EGFP, whose CDS is now after the introduced stop codon. For CRISPR targeting of human *EVC*, the following sgRNA-coding sequences were cloned into pSpCas9(BB)-2A-Puro (PX459) V2.0 (Addgene #62988), as previously described: sgEVC1: cgg​cct​gca​aga​gcg​acg​cg; sgEVC2: cag​ccg​cgc​gtc​gct​ctt​gc; sgEVC3: ctt​tgg​ctt​ggc​tgc​cgc​gc; sgEVC4: gtg​ctg​ctg​ggc​gcc​gcg​ct (Barbeito et al., 2021; Cilleros-Rodriguez et al., 2022). Site-directed mutagenesis was performed by overlap extension PCR as reported previously (Barbeito et al., 2021; Cilleros-Rodriguez et al., 2022). All constructs were validated by Sanger DNA sequencing (Eurofins Genomics).

### 4.4 Immunoprecipitation and Western blot

Immunoprecipitation (IP) and Western blot experiments in HEK293T cells were carried out as previously described (Martin-Hurtado et al., 2019; Barbeito et al., 2021; Cilleros-Rodriguez et al., 2022). Uncropped blots for all the figures are shown in the supplement (Supplementary Figures S7, S8). Briefly, cells were lysed 40–48 h post-transfection, lysate protein concentrations equalized, and IPs performed with FlagM2 agarose or GFP-Trap magnetic agarose beads, as appropriate. After washing the beads, immunoprecipitated proteins were eluted with Laemmli buffer and analyzed by SDS-PAGE using Novex Value Tris-glycine precast gels (Thermofisher). Quantitation of relative band intensities in IP experiments was done as reported before (Cilleros-Rodriguez et al., 2022). For IP of endogenous EVC in MEFs, see interactomics experiments section below. For the endogenous EVC ubiquitination/sumoylation experiment (Supplementary Figure S6), cells were lysed for 30 min at 4°C in buffer containing: 50 mM Tris-HCl pH 7.5, 150 mM NaCl, 1% Igepal CA-630, 20 mM N-ethylmaleimide (from freshly prepared stock), and Halt protease inhibitor cocktail (Thermofisher, #74829). Lysates were then cleared by centrifugation (10 min, 4°C, 17,000 g) and protein concentrations equalized. A pre-clearing step was performed by adding 15 µL/IP of washed Dynabeads Protein G beads (Thermofisher, #10003D) for 1 h at 4°C. After removing the beads, 10 µg of mouse anti-EVC antibody was added per sample for overnight incubation at 4°C (Pacheco et al., 2012). Next day, 50 µL/IP of washed Dynabeads Protein G was added for 3 h at 4°C. The dynabeads were then washed thrice in lysis buffer without protease inhibitors before resuspension in Laemmli buffer and processing for SDS-PAGE and Western blot as above.

### 4.5 Immunofluorescence microscopy

hTERT-RPE1, IMCD3 and MEF cells were grown on coverslips until they reached confluence, and serum-starved for 16–24 h to promote ciliation. Fixation, immunostaining, imaging, and image processing were all as previously reported (Cilleros-Rodriguez et al., 2022). Briefly, cells were fixed with PBS+4% paraformaldehyde (PFA, 5 min, RT), followed by methanol (3 min, −20°C). For MKS1 staining, only methanol was used. After blocking and permeabilization, primary antibodies were added in blocking solution at the above-indicated dilutions (*Reagents and antibodies*). After three washes, secondary antibodies were added together with DAPI to stain DNA. Washed coverslips were then mounted and imaged with a Nikon Ti epifluorescence microscope. Images were processed using Adobe Photoshop and/or Fiji/ImageJ. Signal intensity profiles were obtained with the Plot Profile function of Fiji/ImageJ, also as described (Cilleros-Rodriguez et al., 2022). For HEK293T cells, our protocol was the same, except for two modifications based on recent work: i) coverslips were coated with poly-L-lysine and gelatin; and ii) cells were not serum-starved, as this does not affect their ciliation (Gomez et al., 2022).

### 4.6 Interactomics experiments

#### 4.6.1 Immunoprecipitations

Each of the *n* = 3 independent experiments was performed using fifteen 10-cm plates each of *Evc* ^+/+^ and *Evc* ^*−*/−^ MEFs. Cells were seeded at 1.5 × 10^6^ cells/plate in DMEM+10% FBS and incubated for 1 day before changing medium to DMEM+0.5% FBS, to induce ciliogenesis. After 24 h in this starvation medium, cells were lysed in 1X IP Buffer (Dynabeads CoImmunoprecipitation Kit, Life Technologies, 14321D) with 25 mM NaCl and protease inhibitors. In parallel, 5 mg dynabeads per condition were incubated overnight at 37°C with mouse anti-EVC (30 µg antibody per mg of dynabeads) (Pacheco et al., 2012). Next steps were performed according to the above kit’s manufacturer’s instructions (Life technologies, 14321D). This included overnight 4°C incubation of 5 mg anti-EVC beads with the protein extracts. After two final washes in 25 mM ammonium bicarbonate, the resulting beads were sent to the proteomics facility of the Spanish National Center for Biotechnology (CNB-CSIC).

#### 4.6.2 SDS-PAGE and tryptic digestions

Beads were processed for mass spectrometry as follows: they were eluted with Laemmli buffer and samples loaded into an SDS-PAGE gel (1 mm thick; 4% stacking; 12% resolving), which was run briefly, until the front entered 1 cm into the resolving gel (so the whole proteome was concentrated at the stacking-resolving gel interface). The unresolved protein bands were then visualized with Coomassie, excised, cut into cubes (1 mm^3^), deposited into 96-well plates, and processed automatically in a Proteineer DP (Bruker Daltonics). Digestion protocol was as described (Shevchenko et al., 1996), with minor variations: gel plugs were first washed with 50 mM NH_4_HCO_3_, then with acetonitrile prior to reduction with 10 mM dithiotreitol (DTT) in 25 mM NH_4_HCO_3_ solution. Alkylation was performed in 55 mM iodoacetamide in 50 mM NH_4_HCO_3_. Gel pieces were then rinsed in 50 mM NH_4_HCO_3_, then in acetonitrile, then dried under a nitrogen stream. Digestion was carried out for 4 h at 37°C using 16 ng/μL proteomics-grade Trypsin (Sigma), in 25% acetonitrile in 50 mM NH_4_HCO_3_. Reaction was stopped with 0.5% trifluoroacetic acid in 50% acetonitrile. The eluted tryptic peptides were dried by speed-vacuum centrifugation.

#### 4.6.3 Liquid chromatography and mass spectrometry (LC-ESI-MS/MS)

Each digested sample (1 µg aliquot) was then subjected to 1D-nano LC ESI-MS-MS analysis using a nano liquid chromatography system (Eksigent Technologies nanoLC Ultra 1D plus, SCIEX) coupled to high speed Triple TOF 5600 mass spectrometer (SCIEX) with a Nanospray III source. The analytical column was a silica-based reverse phase Acquity UPLC M-Class Peptide BEH C18 Column (75 μm × 150 mm; 1.7 µm particle size; 130 Å pore size) (Waters). Trap column was a C18 Acclaim PepMapTM 100 (100 μm × 2 cm; 5 µm particle diameter; 100 Å pore size) (Thermofisher), switched online with the analytical column. The loading pump delivered a solution of 0.1% formic acid in water at 2 μL/min. The nanopump provided flow rate of 250 nL/min and was operated with gradient elution. Peptides were separated using a 100 min gradient ranging from 2% to 90% mobile phase B (100% acetonitrile + 0.1% formic acid). Mobile phase A was: 2% acetonitrile + 0.1% formic acid. Injection volume was 5 µL. Data acquisition was performed with the TripleTOF 5600 system. Data were acquired using following parameters: ionspray voltage floating (ISVF): 2300 V, curtain gas (CUR): 35, interface heater temperature (IHF): 150, ion source gas 1 (GS1): 25, declustering potential (DP): 100 V. All data were acquired in information-dependent acquisition (IDA) mode with Analyst TF 1.7 software (SCIEX). For IDA parameters, 250 ms MS survey scan in mass range 350–1250 Da were followed by 35 MS/MS scans of 100 ms in mass range 100–1800 (total cycle time: 4 s). Switching criteria were set to ions with mass-to-charge ratio (m/z) > 350 and (m/z) < 1250, with charge state of two to five, and abundance threshold above 90 counts (cps). Former target ions were excluded for 15 s. IDA rolling collision energy (CE) parameters script was used for automatically controlling CE.

#### 4.6.4 Peptide identification

MS data were processed with PeakView v2.2 software (SCIEX) and exported as mgf files, which were searched using Mascot Server v2.5.1 (Matrix Science) against Uniprot’s *Mus musculus* protein database, together with commonly occurring contaminants. Search parameters were set as follows: enzyme: trypsin; allowed missed cleavages: 2; fixed modification: carbamidomethyl; variable modifications: N-terminal acetyl, pyrrolidone at glutamate and glutamine, and methionine oxidation. Peptide mass tolerance was set to ±25 ppm for precursors and 0.05 Da for fragment masses. Confidence interval for protein identification was 95% (*p* <0.05) and only peptides with individual ion scores above the 1% false discovery rates (FDR) at spectra level were considered correctly identified.

#### 4.6.5 Proteomics data analysis

In each experiment (*n* = 3) and sample (WT *versus* KO), the following parameters were obtained for each identified protein or hit: i) number of unique peptides (#Pept); ii) peptide spectral matches (PSM); iii) percent of protein sequence covered by the identified peptides (% coverage); and iv) sum total of MS ion scores (Protein Score). To analyze these data, we first calculated the WT vs. KO fold change (FC) for all hits. In each experiment, the FC value for each hit “i” was obtained as follows:

$${FC}_{i}={\left(\frac{{PSM}_{WT}+1}{{PSM}_{KO}+1}\right)}_{i}$$

In the above equation, one unit was added to both numerator and denominator to avoid dividing by zero in the many cases where *PSM*_*KO*_ = 0. Thus, FC was calculated as if one additional peptide had been identified in both WT and KO samples, an approach that did not significantly change the final results. We then ranked all hits by mean FC value across the three experiments. All hits with mean FC <3.5 were discarded (leaving 230 hits out of the initial 3733). We then discarded hits with FC <2.0 in two of the experiments, or with FC <2.0 in one experiment if mean FC <5.0. This left 49 hits. For all these, we checked whether there were other entries under the same protein name, as peptides from the same protein were sometimes assigned to different protein IDs. Such redundant entries were combined, and FC values recalculated accordingly, leading to 24 additional discards (one of them, the Y chromosome-linked EIF2S3Y, was discarded after combining its entry with that of EIF2S3X, its 98% identical X-linked counterpart, since it appeared that peptide assignment algorithms had not properly distinguished between the two). After this, we were left with 25 hits, from which we also let go those with FC <3.0 in one or more experiments, leading to the final 14 protein shortlist in Figure 1C. For more details, see Supplementary Data File 1.

To generate the volcano plot in Figure 1B, we calculated mean FC of each hit as above and plotted its binary logarithm in the *x*-axis (log_2_ FC). To calculate *p*-values, an unpaired homoscedastic Student’s t-test was performed comparing, for each hit, its normalized PSM values in WT vs. KO (*n* = 3). The following equation was used to normalize *PSM*_*WT*_ values in each experiment:

$${nPSM}_{WT}=100\cdot \frac{{PSM}_{WT}+1}{\left({PSM}_{WT}+{PSM}_{KO}+2\right)}$$

Above, *nPSM*_*WT*_ is the normalized value, representing *PSM*_*WT*_ as percentage of total PSM for that hit and experiment. To avoid divisions by zero, we again performed all PSM calculations as if one extra peptide had been identified in each sample. An analogous equation was used to calculate *nPSM*_*KO*_. This normalization was undertaken because total numbers of identified peptides (and hence PSM values) varied considerably across experiments. The *p*-values from these t-tests were then graphed in the *y*-axis as −log_10_ (*p*-value). Proteins for which *PSM*_*WT*_ never surpassed one peptide, or for which *PSM*_*WT*_ = 0 in two or more experiments, were not included in the volcano plot. For more details, see Supplementary Data File 1.

## Data Availability

The original contributions presented in the study are included in the article/Supplementary Material. Further inquiries can be directed to the corresponding authors.

## Supplementary material

SUPPLEMENTARY FIGURE S1 Depletion or overexpression of the EVC-EVC2 complex does not affect USP7-GLI1 binding. **(A)** Western blot (WB) of EVC sgRNA CRISPR-Cas9 clones using antibody against human EVC. Parental HEK293T cell lysate (WT) was included as control. **(B)** The indicated constructs were expressed in the WT, 2.1 or c2 cell lines from **(A)**, as shown. Cell lysates were immunoprecipitated (IP) with anti-EGFP beads and analyzed by WB as indicated. Molecular weight markers are on the right. **(C)** The indicated constructs were expressed in wild type HEK293T cells treated with Hedgehog pathway activator SAG (200 nM, 24 hours), or with DMSO as vehicle control. Cell lysates were immunoprecipitated with anti-Flag beads and analyzed by WB as indicated. Molecular weight markers are on the right.

SUPPLEMENTARY FIGURE S2 Chloroquine does not affect endogenous Evc localization in MEFs. *Evc* ^+/+^ MEFs were starved for 16 hours in DMEM + 0.2% FBS in the presence of 10 µM chloroquine or its vehicle (water). Cells were then fixed and stained with the indicated antibodies. Scale bar, 5 µm. No obvious differences were observed between conditions.

SUPPLEMENTARY FIGURE S3 The EVC-EVC2 complex localizes at the EvC zone in hTERT-RPE1 cells. **(A)** EVC-Flag and EVC2-Flag were co-transfected in hTERT-RPE1 cells. After 48 hours, cells were starved for 16 hours in DMEM/F12 + 0.2% FBS before being fixed and stained with the indicated antibodies. Scale bar, 5 µm. Two representative cilia are shown. **(B,C)** Signal intensity for each channel was plotted as a function of distance from the ciliary base for the two cilia in **(A)**. Colors as in **(A)**.

SUPPLEMENTARY FIGURE S4 The EVC-EVC2 complex localizes at the EvC zone in IMCD3 cells. **(A)** EVC-Flag and EVC2-Flag were co-transfected in IMCD3 cells. After 48 hours, cells were starved for 16 hours in DMEM/F12 + 0.2% FBS before being fixed and stained with the indicated antibodies. Scale bar, 5 µm. Two representative cilia are shown. **(B)** Signal intensity for each channel was plotted as a function of distance from the ciliary base for the two cilia in **(A)**. Colors as in **(A)**.

SUPPLEMENTARY FIGURE S5 The exogenous EVC-EVC2 complex localizes throughout the cilium in HEK293T cells. EVC-Flag and EVC2-Flag were co-transfected in HEK293T cells. After 48 hours, cells were fixed and stained with the indicated antibodies. Scale bar, 5 µm.

SUPPLEMENTARY FIGURE S6 Endogenous EVC is not detectably ubiquitinated or sumoylated in MEFs. *Evc* ^+/+^ and *Evc* ^−/−^ MEFs were starved for 16 hours in DMEM + 0.2% FBS in the presence of chloroquine (CQ, 16 hours, 10 µM), MG132 (last 6 hours, 20 µM) or vehicle (DMSO, last 6 hours). Cells were then lysed and lysates subjected to immunoprecipitation (IP) and western blot (WB) analysis with the indicated antibodies. Molecular weight markers are on the right. EVC is detected in *Evc* ^+/+^ MEF IPs as two bands near 130 kDa, but no specific label is observed from these bands with the ubiquitin or SUMO2/3 antibodies.

SUPPLEMENTARY FIGURE S7 Uncropped blots from Figures 2, 3, 4, 6. **(A)** Uncropped blots from Figure 2. **(B)** Uncropped blots from Figure 3. **(C)** Uncropped blots from Figures 4, 6.

SUPPLEMENTARY FIGURE S8 Uncropped blots from Figures 5, 7, 8 and Supplementary Figure S1. **(A)**Uncropped blots from Figure 5. **(B)** Uncropped blots from Figure 7. **(C)** Uncropped blots from Figure 8. **(D)** Uncropped blots from Supplementary Figure S1.
