# Supplementary figures and images for: EVC-EVC2 complex stability and ciliary targeting are regulated by modification with ubiquitin and SUMO
Source: Front Cell Dev Biol. 2023 Jul 27;11:1190258. doi: 10.3389/fcell.2023.1190258 (PMC10413113; doi:10.3389/fcell.2023.1190258)

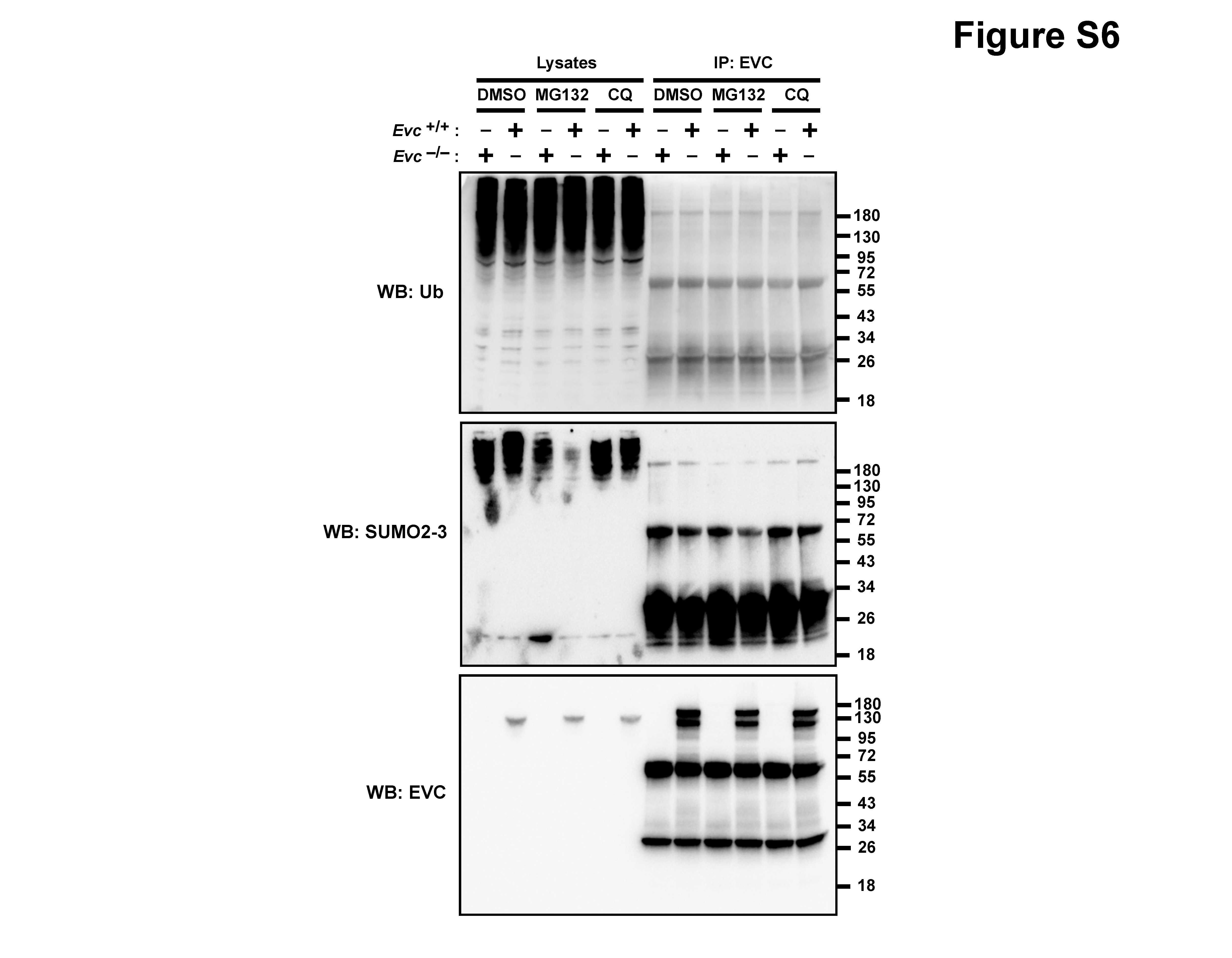

Supplement: Supplementary file 1 [file Image6.TIF]

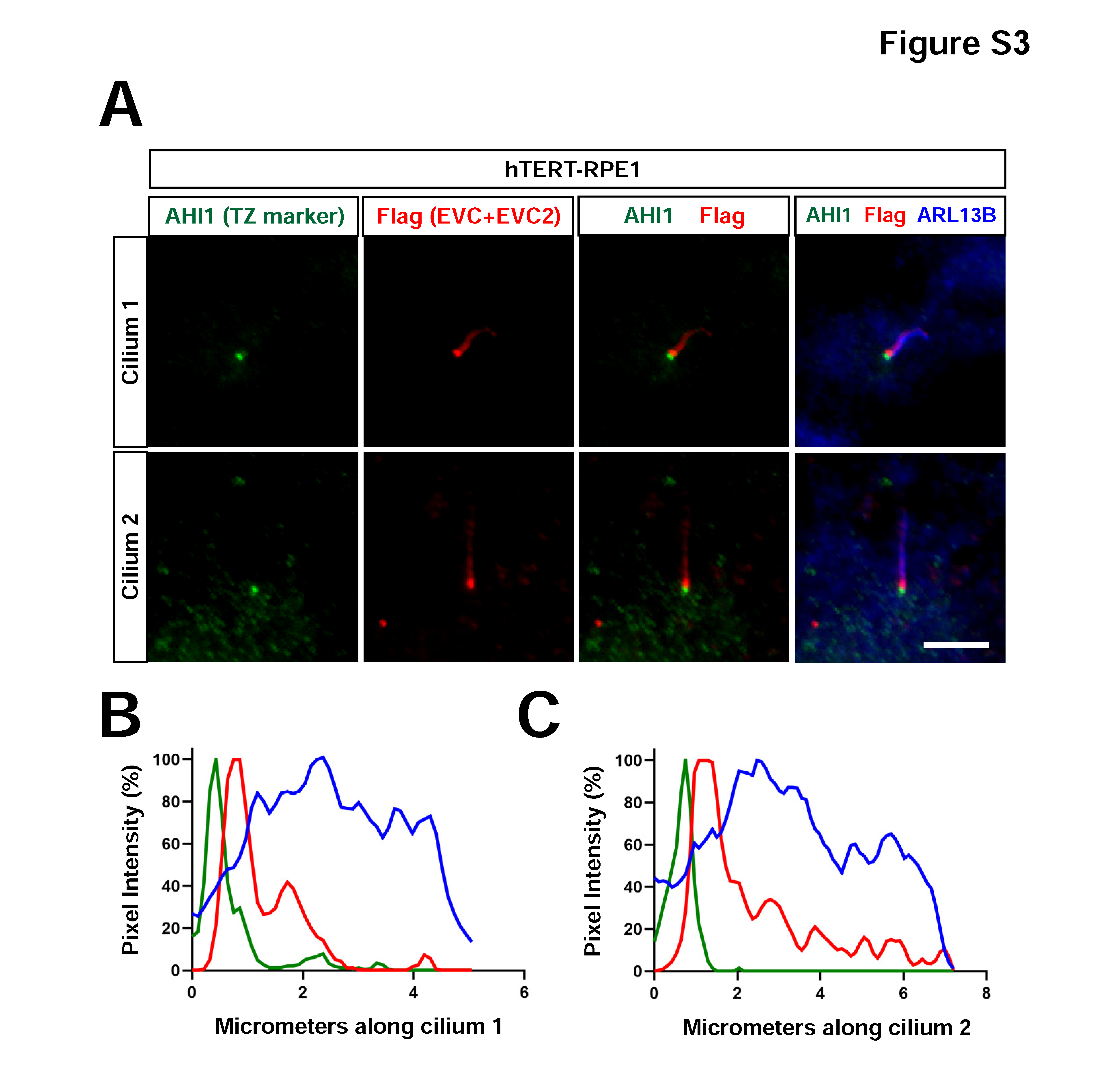

Supplement: Supplementary file 2 [file Image3.TIF]

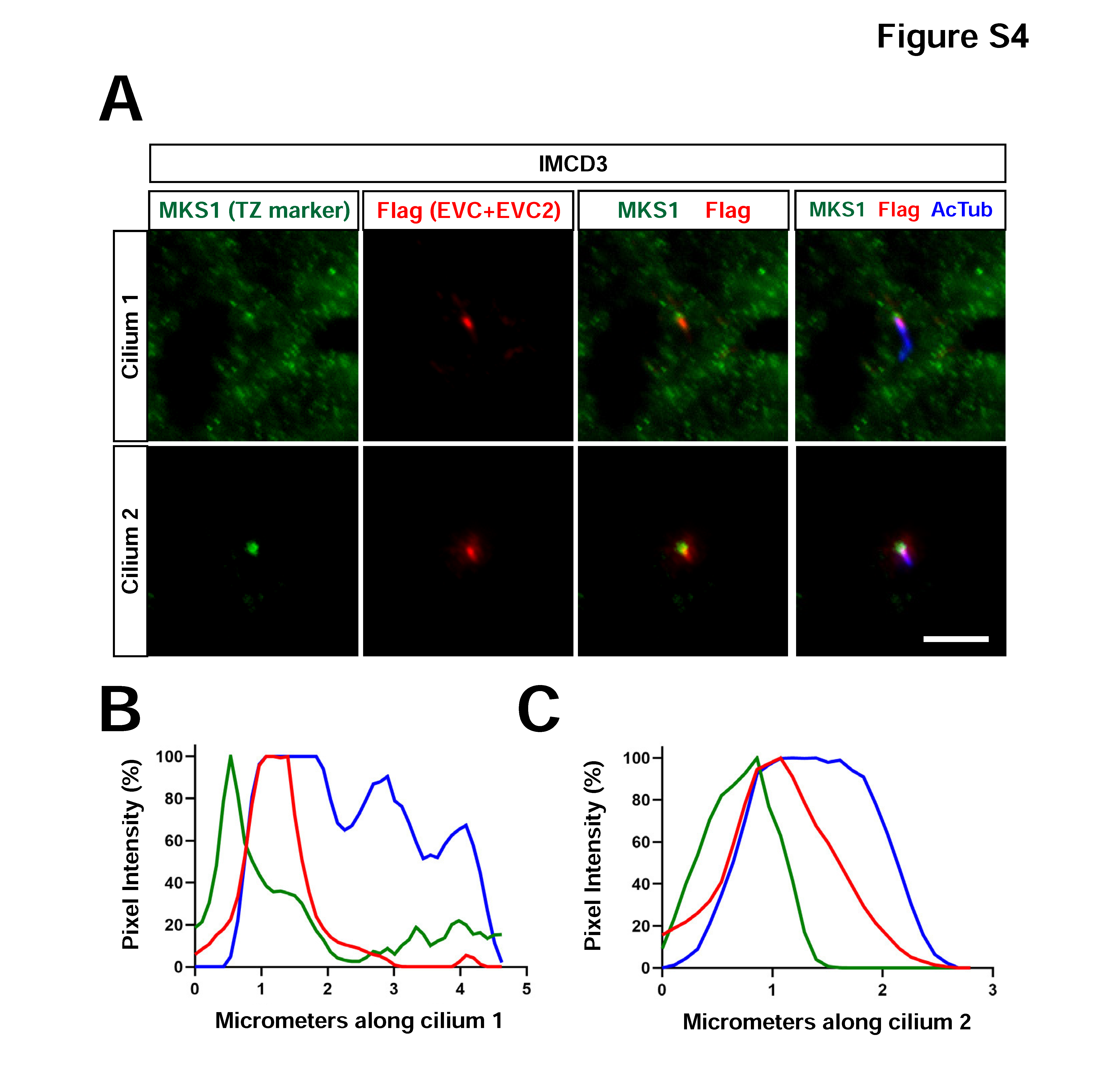

Supplement: Supplementary file 3 [file Image4.TIF]

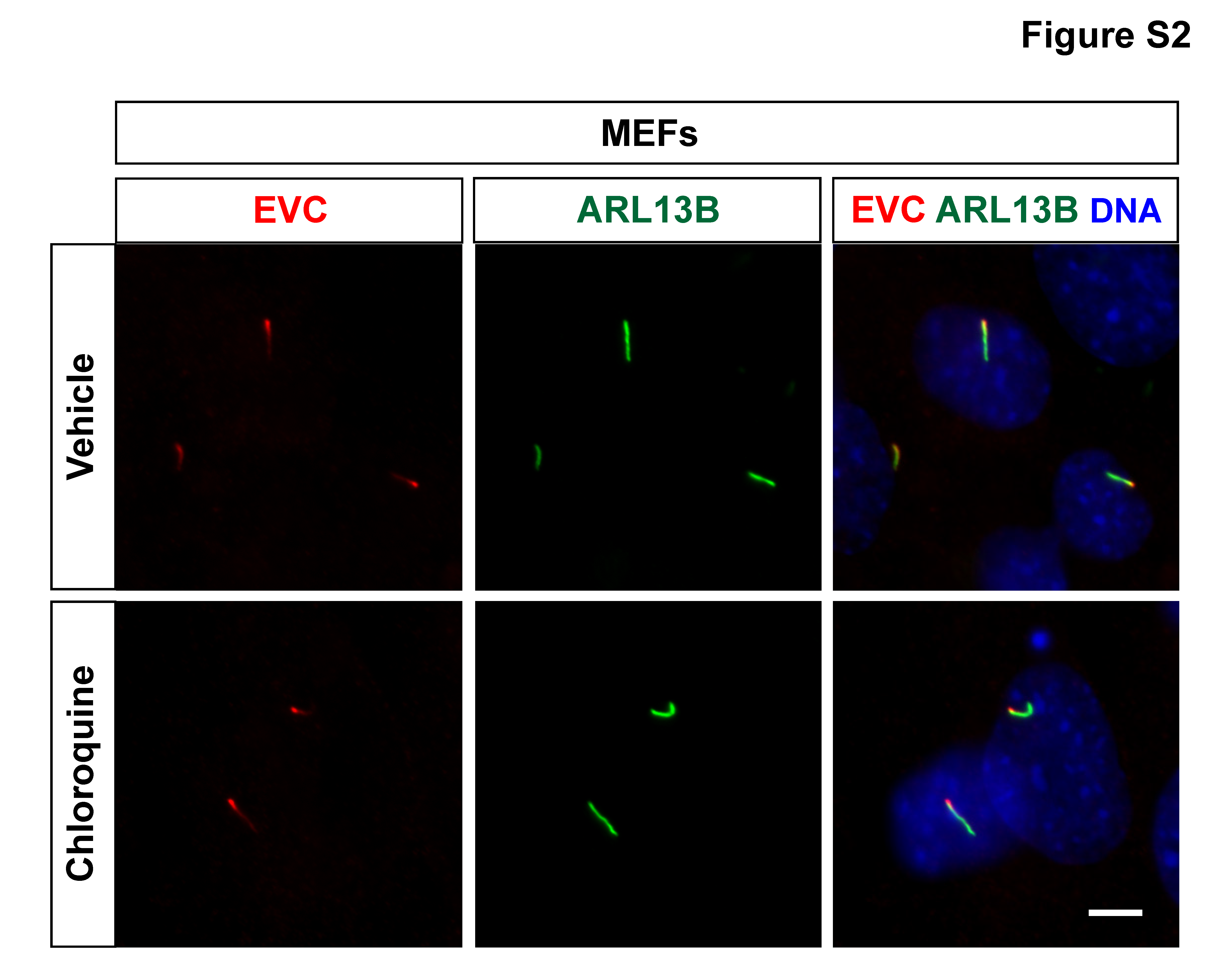

Supplement: Supplementary file 4 [file Image2.TIF]

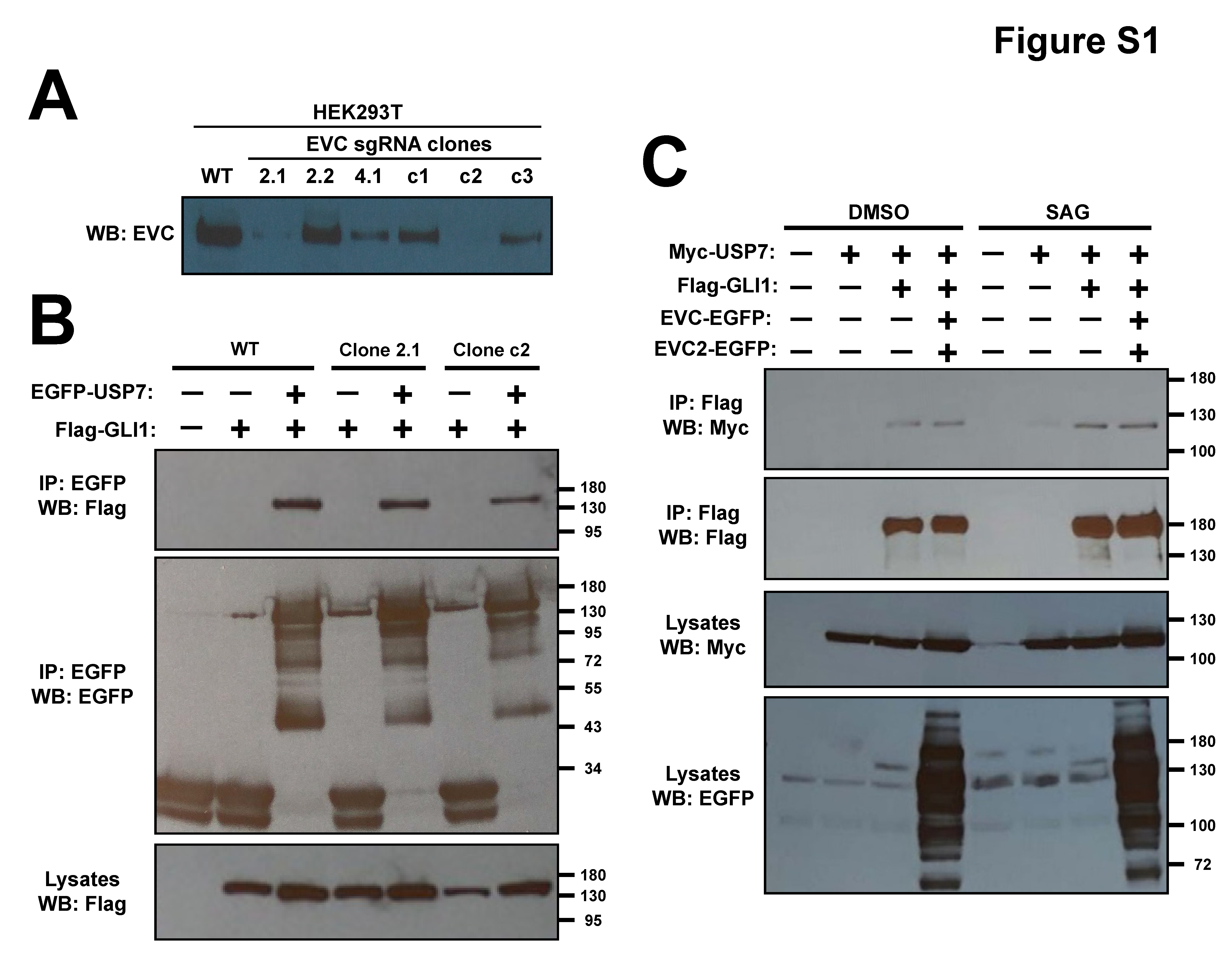

Supplement: Supplementary file 5 [file Image1.TIF]

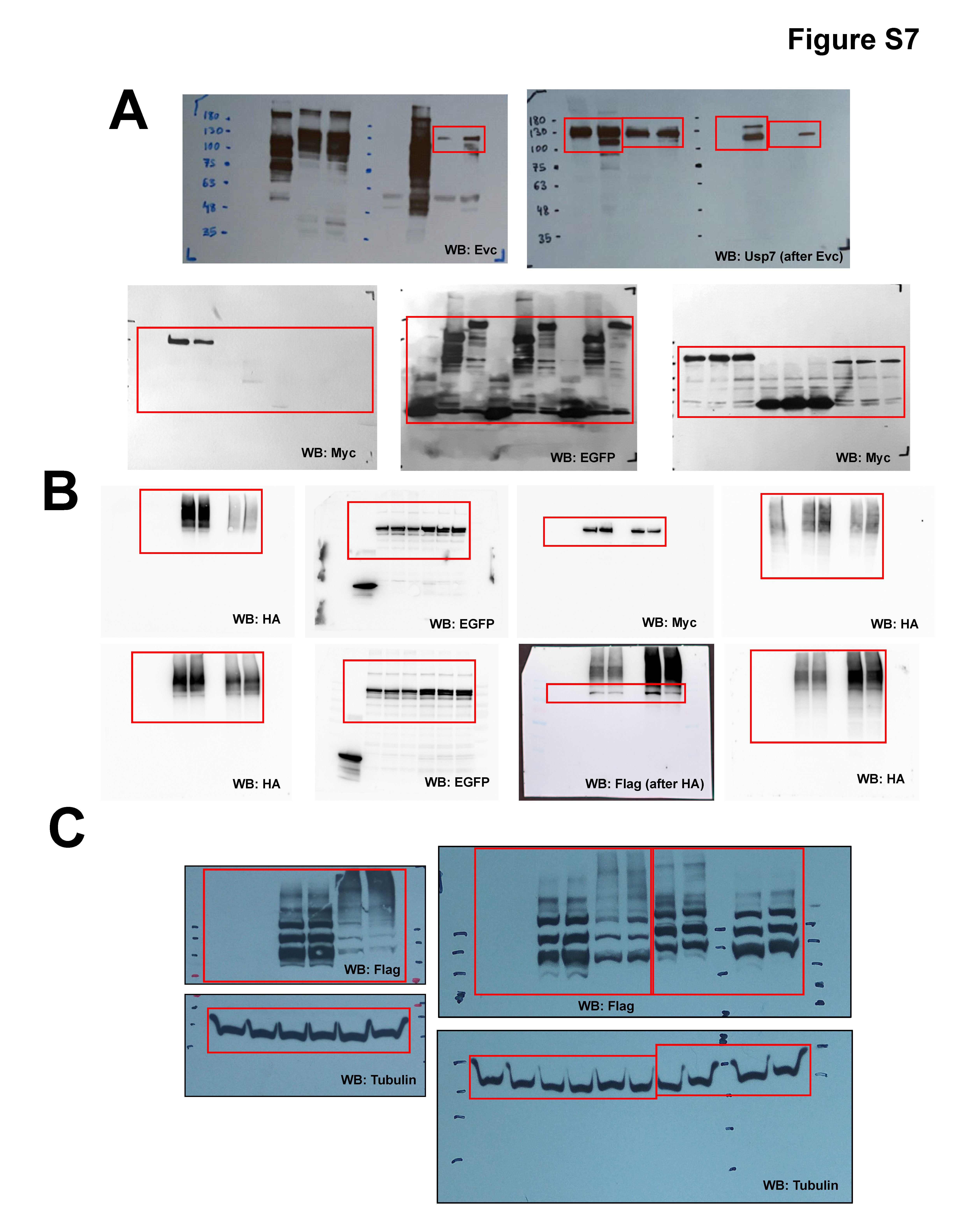

Supplement: Supplementary file 6 [file Image7.TIF]

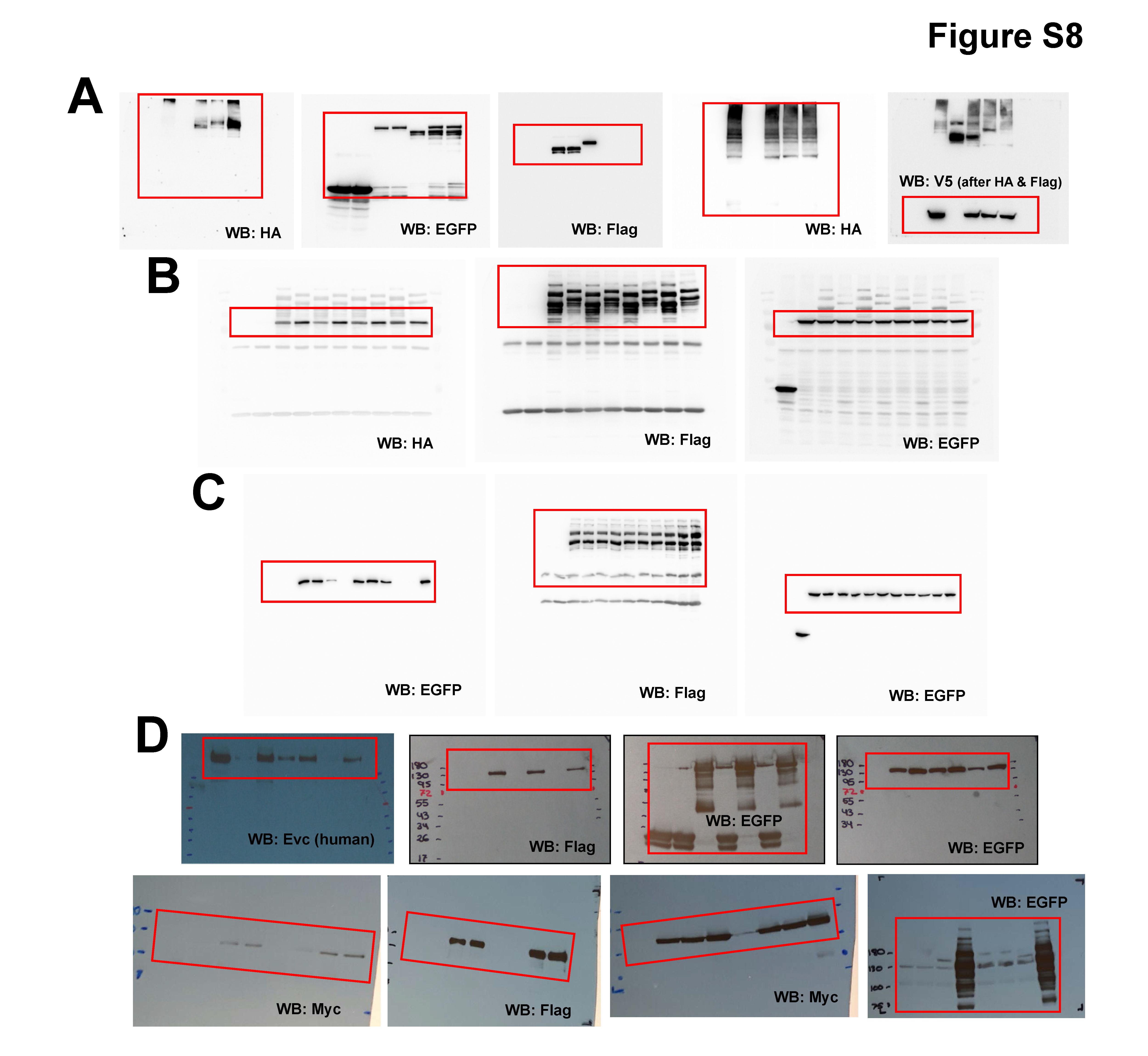

Supplement: Supplementary file 8 [file Image8.TIF]

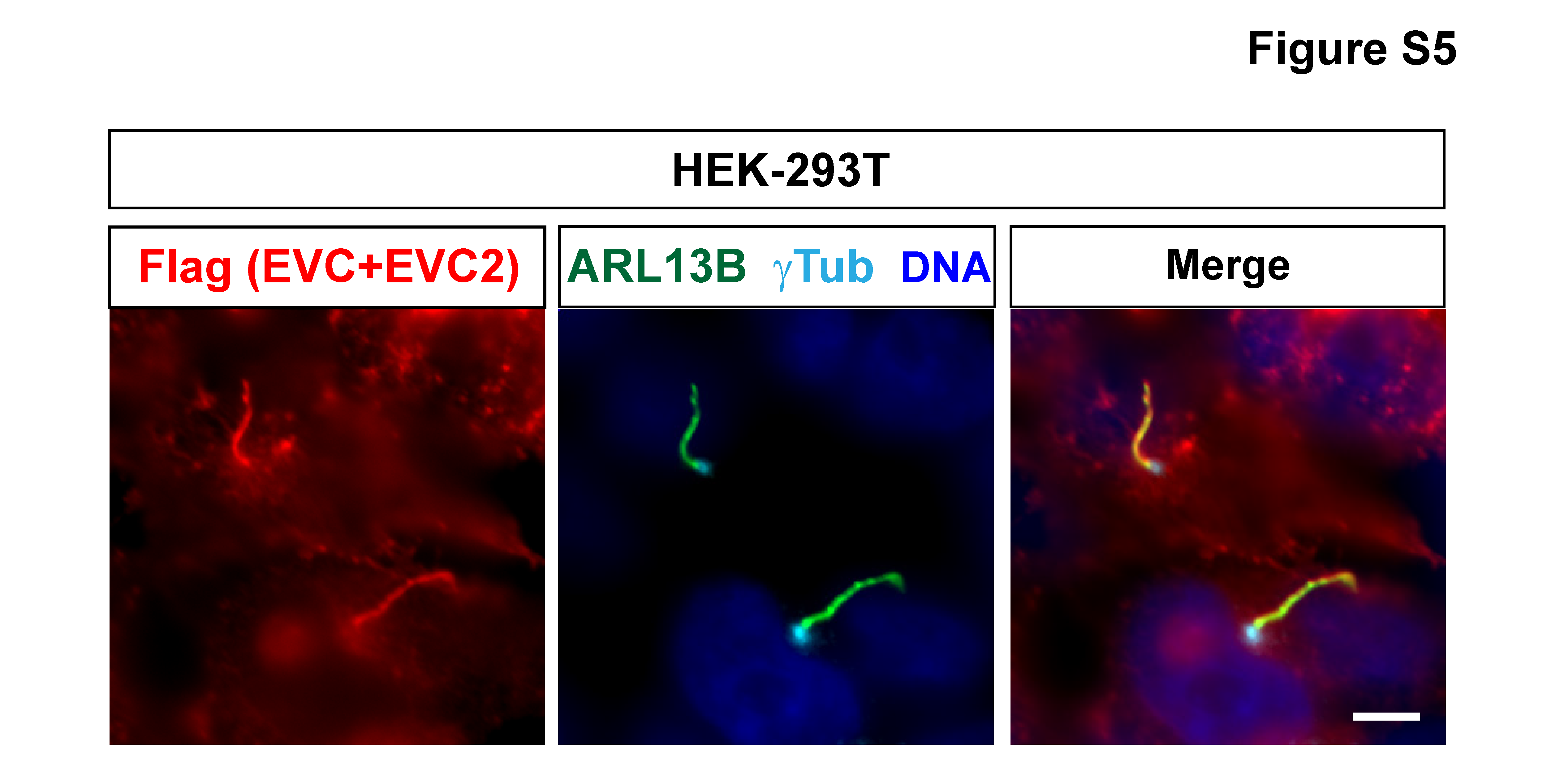

Supplement: Supplementary file 9 [file Image5.TIF]
